# Interfacial water engineering boosts neutral water reduction

**DOI:** 10.1038/s41467-022-33984-5

**Published:** 2022-10-21

**Authors:** Kaian Sun, Xueyan Wu, Zewen Zhuang, Leyu Liu, Jinjie Fang, Lingyou Zeng, Junguo Ma, Shoujie Liu, Jiazhan Li, Ruoyun Dai, Xin Tan, Ke Yu, Di Liu, Weng-Chon Cheong, Aijian Huang, Yunqi Liu, Yuan Pan, Hai Xiao, Chen Chen

**Affiliations:** 1 Engineering Research Center of Advanced Rare Earth Materials, Department of Chemistry, Tsinghua University, 100084 Beijing, China; 2 State Key Laboratory of Chemistry and Utilization of Carbon Based Energy Resources, College of Chemistry, Xinjiang University, 830017 Urumqi, China; 3 College of Materials Science and Engineering, Fuzhou University, 350108 Fuzhou, China; 4 Beijing Advanced Innovation Center for Soft Matter Science and Engineering, Beijing University of Chemical Technology, 100029 Beijing, China; 5 State Key Laboratory of Heavy Oil Processing, China University of Petroleum (East China), 266580 Qingdao, China; 6 School of Electronics Science and Engineering, Center for Public Security Technology, University of Electronic Science and Technology of China, 610054 Chengdu, China

**Keywords:** Electrochemistry, Electrocatalysis, Energy, Catalytic mechanisms

## Abstract

Hydrogen evolution reaction (HER) in neutral media is of great practical importance for sustainable hydrogen production, but generally suffers from low activities, the cause of which has been a puzzle yet to be solved. Herein, by investigating the synergy between Ru single atoms (RuNC) and RuSe_*x*_ cluster compounds (RuSe_*x*_) for HER using ab initio molecular dynamics, operando X-ray absorption spectroscopy, and operando surface-enhanced infrared absorption spectroscopy, we establish that the interfacial water governs neutral HER. The rigid interfacial water layer in neutral media would inhibit the transport of H_2_O*/OH* at the electrode/electrolyte interface of RuNC, but the RuSe_*x*_ can promote H_2_O*/OH* transport to increase the number of available H_2_O* on RuNC by disordering the interfacial water network. With the synergy of RuSe_*x*_ and RuNC, the resulting neutral HER performance in terms of mass-specific activity is 6.7 times higher than that of 20 wt.% Pt/C at overpotential of 100 mV.

## Abstract (web summary)

Understanding the slow kinetics of hydrogen evolution reaction in neutral media is of fundamental importance for the rational design of high-performance electrocatalysts for hydrogen energy. Here, by studying Ru single atom and RuSe_*x*_ cluster, the authors report how the rate of hydrogen evolution reaction activity in neutral media is governed by interfacial water.

## Introduction

In the background of global consensus on decarbonization, electrochemical water splitting in neutral conditions is expected to play a major role in the “green hydrogen economy” in the long run^1–3^. However, as the central component in water splitting, the electrocatalysts for hydrogen evolution reaction (HER) commonly perform less effectively in neutral media than in alkaline ones, although HER in both conditions undergoes the same reaction pathways. The insufficient understanding on the underpinning mechanism has been severely hindering the design and development of high-performance catalysts for neutral HER^4^.

For HER in neutral and alkaline electrolytes, H_2_O is the primary source of protons. A complete HER cycle involves the catalytic water dissociation (H–OH bond cleavage) on the electrode surface, and the transport of related intermediates (H_2_O*/OH*) at the electrode/electrolyte interface^5,6^; the latter, as shown by some recent evidences, play a critical role in the overall kinetics^7–9^. As the pH increases, the flexibility of the H-bonded water layer at the interface decreases, which obstructs the intermediates to penetrate the interfacial water layer to diffuse into the bulk solution, thereby suppressing the regeneration of surface active sites^10–13^. In alkaline media, the non-specifically adsorbed OH^–^ anions could promote the intermediates to go through the interfacial water layer^14,15^. Yet unfortunately, in neutral media, OH^–^ ions barely exist, and thus it is rather difficult for the intermediates to diffuse into the bulk solution owing to the rigid interfacial water layer^16^. This probably explains the fact that electrocatalysts that could effectively catalyze the water dissociation in alkaline media could rarely give high performances in neutral electrolytes.

To verify the critical role of interfacial water in neutral HER, one needs to employ an ideal catalyst model that could decouple the effect of interfacial water from the step of catalytic water dissociation. As a high-performance and cost-competitive HER catalyst, nitrogen-coordinated Ru single atoms on graphitic carbon (denoted as RuNC) have recently been reported to efficiently catalyze the water dissociation^17,18^; in addition, the positively charged Ru centers supported on hydrophobic graphitic carbon inherently do not favor the transfer of H_2_O*/OH* between the interfacial water layer and active centers. More importantly, benefiting from the well-characterized structure of active centers, RuNC would serve as an ideal platform for the study on interfacial water^19^.

Although it is still a great challenge to regulate the interfacial water, some works have reported that water molecules could be reoriented on negatively charged surfaces^20,21^. In particular, the chalcogen elements irreversibly adsorbed on noble metals have been found to function as electron acceptors to reorient the water molecules^22,23^; such structures are quite similar to the core–shell RuSe_*x*_ cluster compounds, whose HER performance are yet to be investigated^24^. Here we predict that the RuSe_*x*_ could be an effective additive for RuNC to regulate the interfacial water structure, as the Ru core could act as an electron reservoir to transfer electrons to the Se shell.

In this work, we employ density function theory (DFT) calculations to assess the rationality of using RuNC as the platform for the study on interfacial water; we also conduct ab initio molecular dynamics (AIMD) calculation to estimate the feasibility of using RuSe_*x*_ to regulate the interfacial water. Then we prepare the composite catalyst RuSe_*x*_–RuNC in a controllable manner, and investigate the behavior of interfacial water structure in neutral HER. By analyses of operando attenuated total reflectance–surface-enhanced infrared absorption spectroscopy (ATR-SEIRAS) and operando X-ray absorption spectroscopy (XAS), we find that the interfacial water layer in neutral media is more rigid than in acidic and alkaline media, and the rate of neutral HER is governed by interfacial water. Significantly, RuSe_*x*_ could impair the rigid network of interfacial water layer in neutral media, thereby accelerating the transport of H_2_O*/OH* to increase the number of available H_2_O* on neighboring RuNC, and thus dramatically enhancing the activity for neutral HER (with an overpotential of merely 29 mV at 10 mA cm^–2^). Our approach here employs a composite material featuring the atomic interface between single atoms and cluster compounds to regulate the structure of interfacial water layer, and opens up other avenues for the design and development of next-generation electrocatalysts.

## Results

### DFT and AIMD theoretical simulation

The DFT and AIMD calculation were carried out to simulate the catalytic pathway and the interfacial water, respectively^25,26^. According to previous calculation results, three different theoretical models were constructed (Supplementary Fig. 1). For metallic Ru, we employed a model with the lowest-energy Ru(001) facets exposed; for RuSe_*x*_, we employed a model with Se atoms adsorbed on the hexagonal close-packed (*hcp*) hollow sites of Ru(001) (denoted as RuSe_*n*_, *n* corresponding to the Se coverage)^27^; for RuNC, we employed a model featuring the thermodynamically most stable MN_4_ (M = metal) structure^28^.

**Fig. 1:**
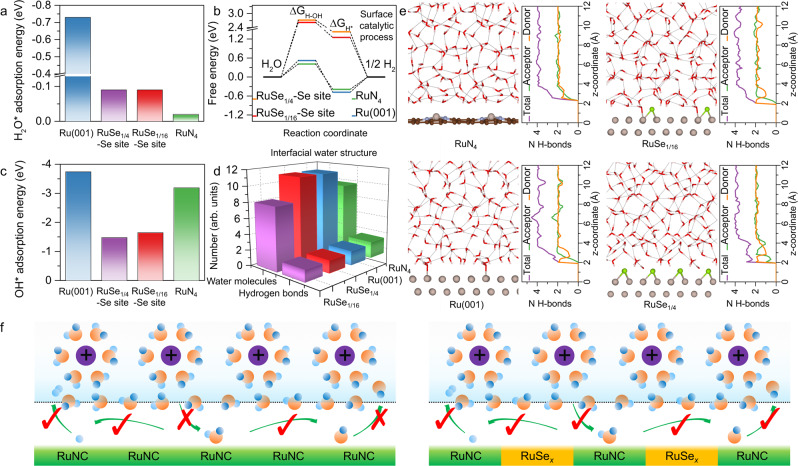
Theoretical simulation. **a** The adsorption energies of water molecule for different models. **b** The free energy changes of water dissociation and the Gibbs free energies of hydrogen adsorption for different models. **c** The adsorption energies of hydroxide for different models. **d** The average number of hydrogen bonds per interfacial water molecule and the corresponding number of water molecules for different models. **e** The representative snapshots of the structure of interfacial water molecules and the corresponding average distribution of hydrogen bond number along the surface. **f** Proposed mechanisms for neutral HER over RuNC (left) and RuSe_*x*_–RuNC (right).

The surface catalytic HER process in neutral condition was simulated by calculating the related parameters for the steps of water adsorption, water dissociation, hydrogen desorption, and hydroxide desorption. Compared with Ru(001), RuSe_1/4_, and RuSe_1/16_, RuN_4_ shows the weakest H_2_O* adsorption energy (*E*_H2O*_, −0.02 eV), suggesting that it is difficult for RuN_4_ to drive water molecules to go through the interfacial water layer to approach the active sites (Fig. 1a and Supplementary Fig. 2). However, the lowest free energy change of water dissociation (Δ*G*_H–OH_, 0.41 eV) on RuN_4_ confirms that once water molecules arrive at the active sites, they can be efficiently dissociated, which may be due to the unique water dissociation pathway on single Ru atom centers (Fig. 1b and Supplementary Fig. 3 and 4). After water dissociation, the resulting H* reacts with another H_2_O (or another H*) to generate H_2_, and the resulting OH* desorbs from active site to go through the interfacial water layer to complete the entire surface catalytic process. Because of the strong oxygen affinity of Ru element^29^, Ru(001) and RuN_4_ models show strong adsorption of OH*, suggesting that the active sites would be blocked by OH* (Fig. 1c and Supplementary Fig. 5). Noticeably, the calculated free energy of single hydrogen adsorption (Δ*G*_H*_) on RuN_4_ is −0.39 eV (Supplementary Fig. 6). Meanwhile, the formation of two-hydrogen intermediates can also be observed on RuN_4_, and the free energy change for the adsorption of the second hydrogen (Δ*G*_H2*_) on RuN_4_ is −0.02 eV (Supplementary Fig. 7). Both Δ*G*_H*_ and Δ*G*_H2*_ are close to zero, i.e., that the formation of H intermediates is thermoneutral, indicating a balance between H* transfer and H_2_ removal on RuN_4_^30^. Therefore, although from the view of surface reaction kinetics, RuN_4_ can accelerate the surface catalytic HER process, from the view of thermodynamics, RuN_4_ would inhibit the transfer of H_2_O*/OH* between the interfacial water layer and active centers.

As a low *E*_OH*_ value is usually correlated with a small Δ*G*_H–OH_ value, it is unlikely to weaken OH* adsorption while maintaining high-efficiency water dissociation by simply regulating the electronic structure of RuN_4_. For studying H_2_O*/OH* transport from other view of interfacial water engineering, the number of water molecules and hydrogen bonds at the interfacial region was further analyzed^31^. The decreased average number of hydrogen bonds per water molecule within interfacial region results in a less ordered interfacial water network, allowing for a more facile intermediate transfer between the interfacial region and the bulk solution. According to the AIMD calculations, the average number of hydrogen bonds per interfacial water molecule for different models follows the order of RuSe_1/4_ (1.5) < RuSe_1/16_ (1.6) < Ru(001) (1.8) < RuN_4_ (2.1), and the corresponding numbers of water molecules are 8, 11, 11, and 9, respectively (Fig. 1d). The representative snapshots and the average distributions of hydrogen bond number confirm that the interfacial water molecules stay farther from the surface of RuN_4_ than from that of Ru(001), RuSe_1/4_, and RuSe_1/16_ (Fig. 1e), which may be caused by the hydrophobicity of graphene substrates^32^. The interfacial water molecules on RuN_4_ behave an O-down configuration. Each water molecule in the O-down configuration donates ~2H atoms and accepts ~1H atom, forming an ordered network. Although Ru(001) can promote the H_2_O ⇄ H_2_O* interconversion by water chemisorption, the chemisorbed water molecule would donates ~2H atoms and form strong hydrogen bonds with neighboring water molecules^33^, disfavoring the OH* ⇄ OH^–^ process. Interestingly, when the Se coverage is increased to 1/4, water molecules cannot be adsorbed on Ru atoms. Meanwhile, the increase of Se coverage can effectively disorder the interfacial water network of Ru(001) by relocalizing the surface electrons on Se atoms (Supplementary Fig. 8). The electron-rich environment on the Se atoms drives a change in the conformation of the interfacial water molecule from O-down to H-down, resulting in a significant decrease in the H donors and acceptors^20–23,34^. Compared with RuN_4_, Ru(001), and RuSe_1/16_, RuSe_1/4_ with the most disordered interfacial water network structure would promote both H_2_O ⇄ H_2_O* and OH* ⇄ OH^–^ processes, indicating that although RuSe_*x*_ cannot accelerate the surface catalytic HER process, it can optimize the flexibility of interfacial water layer for H_2_O*/OH* transport. Therefore, effectively decoupling the interfacial water from the surface catalytic HER process can be achieved by the combination of Ru single atom and RuSe_*x*_ cluster compounds (Fig. 1f).

### Material synthesis and characterization

Encouraged by the above theoretical results, we developed an organic–inorganic multi-conversion method to prepare the RuSe_*x*_–RuNC composite. RuSe_*x*_ was prepared by direct conversion of Ru-doped [ZnSe](DETA)_0.5_ (Ru-[ZnSe](DETA)_0.5_, diethylenetriamine, DETA) featuring Ru…Se binding, and RuNC was prepared from Ru(acac)_3_@ZIF-8 (zeolite imidazolate frameworks-8, ZIF-8) with Ru…O binding. To ensure the close contact between RuSe_*x*_ and RuNC, the distances between Ru…Se and Ru…O binding were adjusted by the topological conversion of ZIF-8 from [ZnSe](DETA)_0.5_. In a typical synthesis, uniform [ZnSe](DETA)_0.5_ nanobelts were first grown on one side of the carbon paper by a seed method (Supplementary Fig. 9). Ru species could be easily introduced into the [ZnSe](DETA)_0.5_ to form Ru…Se binding because of the similar ionic radii of Zn^2+^ (74 pm) and Ru^3+^ (68 pm) (Supplementary Fig. 10). Then, the Ru-[ZnSe](DETA)_0.5_ served as a sacrificial template providing edged Zn nodes for nucleation and growth of ZIF-8 (Supplementary Fig. 11). Ru(acac)_3_ with Ru…O binding is trapped in the micropores of derived ZIF-8 nanorods^35^. The ligand exchange reaction between imidazolate and diethylenetriamine facilitates [ZnSe](DETA)_0.5_ to gradually collapse within 6 h, which drives Ru…Se binding to be uniformly distributed into ZIF-8 along with Ru…O binding (Supplementary Figs. 12–14). Afterward, the derived ZIF-8 nanorods with Ru…Se and Ru…O binding were pyrolyzed at 800 °C under Ar flow, during which the Ru…O binding were reduced to RuNC in situ by carbonization of the organic linker. In contrast, the reducing ability of the system is not enough to reduce Ru…Se binding (probably because of the low affinity between C and Se), and therefore the Ru…Se binding aggregated into RuSe_*x*_^36^.

**Fig. 2:**
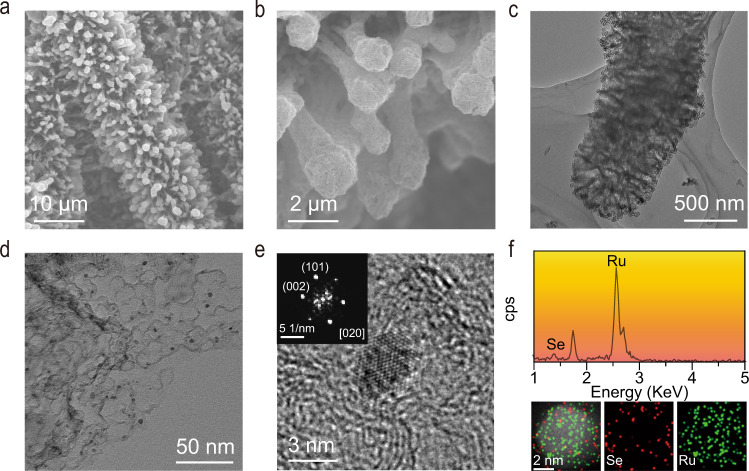
Characterizations on the morphology and microstructure of ReSe_*x*_–RuNC. **a**, **b** SEM images. **c**, **d** TEM images. **e** HRTEM image and corresponding FFT pattern. **f** HAADF-STEM images and element analysis by STEM-EDX.

Field-emission scanning electron microscopy (FESEM) images show that a highly porous three-dimensional ordered array architecture was formed after heating (Fig. 2a, b). The structural units of the ordered array architecture are constructed from uniformly distributed carbon spheres (Supplementary Fig. 15), which attributed to ZnSe_*x*_ evaporation (Supplementary Fig. 16). The specific Brunauer–Emmett–Teller surface area of the architecture was determined to be 782.1 m^2^ g^−1^, which is significantly higher than those of the commercial Pt/C and Ru/C catalyst (Supplementary Fig. 17). Bright‐field transmission electron microscopy (TEM) images show that the architecture consists of RuSe_*x*_ nanoparticles (RuSe_*x*_ NPs mode size value ~4.7 ± 0.5 nm) evenly distributed within a hollow carbon sphere network (carbon spheres mode size value ~17.5 ± 0.5 nm) (Fig. 2c, d and Supplementary Fig. 18). Significantly, we noted that the RuSe_*x*_ NPs are not encapsulated by a shell of graphitic carbon, but are firmly immobilized by hollow carbon spheres. This micro/nano structure ensures that RuSe_x_ can not only be directly exposed to the electrolyte, but also withstand violent gas evolution. The crystalline structure of the RuSe_*x*_ was studied with high-resolution TEM (HRTEM), in combination with the corresponding fast-Fourier transform (FFT) pattern. As revealed in the HRTEM image for an individual NP, the interplanar crystal lattice spacings are 0.20 and 0.21 nm, which can be attributed to the {101} and {002} facets of *hcp* Ru, respectively, viewed along the <020> zone axis (Fig. 2e). Powder X-ray diffraction (XRD) patterns also show a series of Bragg reflections corresponding to the diffractions from the *hcp* Ru (JCPDS card no. 06-0663) (Supplementary Fig. 19). Significantly, the energy-dispersive X-ray (EDX) analysis under high-angle annular dark-field scanning TEM (HAADF-STEM) mode indicates the coexistence of Se and Ru species on an individual NP (Fig. 2f). The element mapping images show that the Ru species is uniformly distributed throughout the entire of NP, whereas the Se species is segregated at the edges. The corresponding Ru/Se atomic ratio is 97.3/2.7, in good agreement with the Ru/Se atomic ratio of 96.9/3.1 obtained via inductively coupled plasma optical emission spectrometry (ICP-OES) (Supplementary Fig. 20).

**Fig. 3:**
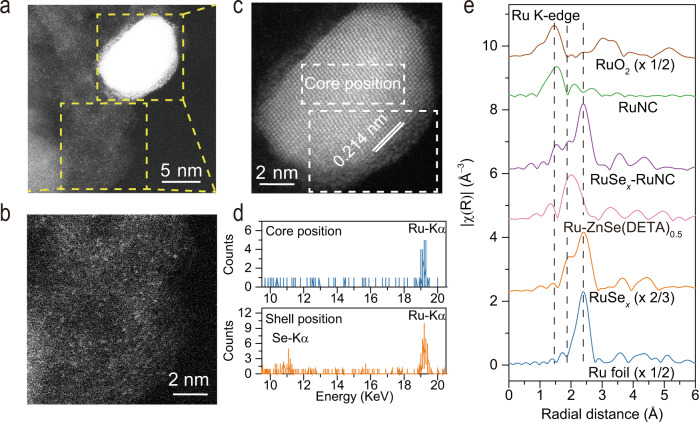
Fine structure characterization. **a**–**c** The sub-Å resolution HAADF-STEM images of RuSe_*x*_ and surrounding RuNC. **d** The element analysis by sub-Å resolution HAADF-STEM-EDX. **e** The *k*^2^-weighted FT spectra of Ru *K*-edge EXANES.

Further information on the atomic-level structure of the RuSe_*x*_ was obtained via aberration-corrected HAADF-STEM. As revealed by the sub-Å resolution HAADF-STEM image for an individual NP (Fig. 3a–c), the outermost atom columns with dark contrast exhibit an amorphous shell structure, whereas the underlying core atom columns with bright contrast exhibit a single-crystalline structure. The difference between the EDX spectra at core and shell positions marked on the sub-Å resolution HAADF-STEM image provides direct evidence for the formation of a core/shell RuSe_*x*_, in which the core is *hcp* crystalline Ru oriented along the [002] direction, and the outermost surface of Ru atoms is uniformly covered by amorphous Se atoms with a lamellar morphology (Fig. 3d and Supplementary Figs. 21 and 22). Noticeably, in addition to RuSe_*x*_, Ru species also exists in RuNC, which can also be readily distinguished within the carbon support around the RuSe_*x*_ NPs. To precisely probe the short-range structure of RuSe_*x*_ NPs and the local coordination environment of RuNC, X-ray absorption spectroscopy (XAS) was carried out. As observed in the Ru *K*‐edge XANES (X-ray absorption near-edge structure) spectra (Supplementary Fig. 23), the comparison of RuNC and RuSe_*x*_ with Ru foil and RuO_2_ references imply that the Ru in RuSe_*x*_ is only partially oxidized and mainly in metallic state, whereas the Ru single atoms in RuNC are positively charged, which agrees well with previous results^37,38^. Notably, the Ru *K*-edge position and the white line intensity of RuSe_*x*_–RuNC are between those of RuSe_*x*_ and RuNC, implying the combined contributions from RuSe_*x*_ and RuNC. The Fourier-transform (FT) *k*^2^‐weighted function of extended XAFS (FT-EXAFS) spectra (Fig. 3e and Supplementary Fig. 24) revealed that the RuNC exhibits one peak at 1.53 Å, attributed to the scattering of the Ru–N(O), whereas the RuSe_*x*_ exhibits two peaks at 1.95 and 2.40 Å, attributed to the scattering of Ru–Se and Ru–Ru, respectively (without phase correction). For RuSe_*x*_–RuNC, the Ru–N(O), Ru–Se, and Ru–Ru scattering paths further confirmed that the coexistence of RuSe_*x*_ and RuNC. Interestingly, benefiting from the good generality of our synthesis method, a series of RuNC samples and/or RuSe_*x*_ samples with varied Ru contents, as well as a RuSe_*x*_–RuNP sample, were prepared (Supplementary Tables 1 and 2 and Fig. 25).

### Electrochemical performance evaluation

**Fig. 4:**
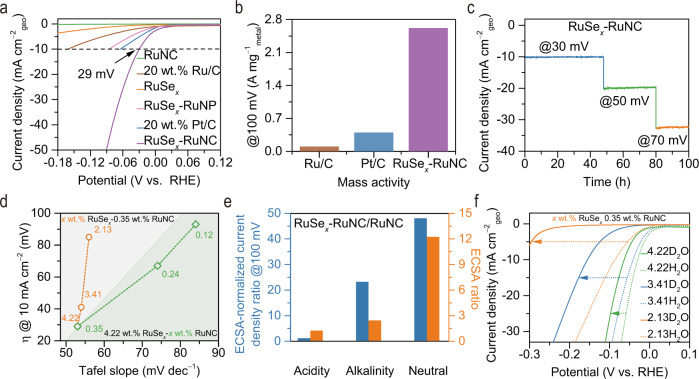
Electrochemical performance. **a** The linear sweep voltammetry curve per geometric area. **b** The mass activity of commercial Ru/C, commercial Pt/C, and RuSe_*x*_–RuNC. **c** The chronopotentiometric curves per geometric area. **d** The comparison of overpotential and Tafel slope per geometric area. **e** The comparison of ECSA and ECSA-normalized current density. **f** The kinetic isotope effect measurements.

The electrochemical tests were performed in H_2_-saturated 1.0 M KPi (potassium phosphate buffer solution, pH = 7) at room temperature using a standard three-electrode system. The HER performance of RuSe_*x*_–RuNC was assessed by linear sweep voltammetry. The geometric area (geo) normalized polarization curves (90% iR‐correction, the details of iR‐correction shown in Supplementary Fig. 26) show that the overpotential over RuSe_*x*_–RuNC (Ru loading 22 μg cm^–2^) is only 29 mV (vs. reversible hydrogen electrode, RHE, the details of calibrating reference electrode shown in Supplementary Fig. 27) to deliver a current density of 10 mA cm^–2^, which is much lower than those of the comparison samples (RuSe_*x*_, RuNC, RuNP–RuSe_*x*_, and 5 wt.% Ru/C with 40 μg cm^–2^ Ru loading) and the commercial 20 wt.% Pt/C (Pt loading 40 μg cm^–2^) (Fig. 4a). Such an activity in terms of overpotential is comparable or even superior to those of previously reported electrocatalysts (Supplementary Table S3). The gas products during HER were determined by an on-line gas chromatography analysis system and the Faradaic efficiency of H_2_ is close to 100 % (Supplementary Table S4). In addition, the RuSe_*x*_–RuNC exhibits a higher mass activity of 2.61 A mg^–1^ at an overpotential of 100 mV, which is 6.7 times greater than that of commercial 20 wt.% Pt/C (Fig. 4b). Notably, the current densities over RuSe_*x*_–RuNC show barely any decline over 100 h of continuous operation, in sharp contrast to the rapid deactivation of RuSe_*x*_ and RuNP–RuSe_*x*_ within 4 h (Fig. 4c and Supplementary Figs. 28 and 29). SEM, TEM, EDX, and X-ray photoelectron spectroscopy (XPS) studies revealed no distinct changes in morphology or chemical composition for the used RuSe_*x*_–RuNC (Supplementary Figs. 30–32).

To investigate the different roles of RuSe_*x*_ and RuNC for the superior activity in neutral HER, we conducted neutral HER tests with a series of RuSe_*x*_–RuNC samples with different RuSe_*x*_ and RuNC contents (Fig. 4d and Supplementary Figs. 33 and 34). The Tafel slope of pristine RuSe_*x*_ (4.22 wt.% Ru) is 198 mV dec^–1^, whereas the Tafel slope is lowered to 84 mV dec^–1^ by incorporating RuNC (0.12 wt.% Ru) and RuSe_*x*_ (4.22 wt.% Ru) at the same time, suggesting that the rate‐determining step changes from the Volmer step (H_2_O* + e^−^ ⇄ H* + OH^−^) to the Heyrovsky step (H* + H_2_O + e^−^ ⇄ H_2_ + OH^−^)^39–43^. When the RuNC content increases from 0.12 to 0.35 wt.% while the RuSe_*x*_ content is kept at 4.22 wt.%, the Tafel slope is lowered from 84 to 53 mV dec^–1^, and the resulting overpotential is lowered from 93 to 29 mV at 10 mA cm^–2^, indicating that RuNC can promote H_2_O*/H* activation. In contrast, when the RuSe_*x*_ content increases from 2.13 to 4.22 wt% while the RuNC content is kept at 0.35 wt.%, the overpotential is lowered from 85 to 29 mV, but there is no significant further decrease in the Tafel slope. This indicates that changes in RuSe_*x*_ content do not cause significant changes in the rate-limiting step of the neutral HER, which may be related to the chemically inert of H* activation on RuSe_*x*_.

The intrinsic activity of RuSe_*x*_ was further investigated by comparing the normalized polarization curves of RuSe_*x*_–RuNC, RuSe_*x*_, and RuNC by electrochemical active surface area (ECSA) in acidic, neutral, and alkaline media (Fig. 4e and Supplementary Figs. S35–S40). After ECSA normalization, RuSe_*x*_ shows a negligible activity at all applied potentials in all media, confirming that RuSe_*x*_ is chemically inert for H_2_O*/H* activation, in good agreement with theoretical calculation results. In addition, it is worth noting that the ECSA of RuNC in neutral media is far smaller than those in alkaline and acidic media, indicating that although RuNC can activate H_2_O*/H*, it has a poor ability to host H_2_O* in neutral media. Interestingly, with the assistance of RuSe_*x*_ to RuNC, the ECSA of RuSe_*x*_–RuNC is 12.2 times, 2.4 times, and 1.2 times higher than that of RuNC in neutral, alkaline, and acidic media, respectively, indicating that RuSe_*x*_ can significantly enhance the available H_2_O* on RuNC in neutral media. Moreover, the ECSA-normalized current density of RuSe_*x*_–RuNC are 47.9 and 23.1 times higher than that of RuNC in neutral and alkaline media, respectively, and similar to that of RuNC in acidic. Therefore, the improvement of HER performance of RuNC by the assistance of RuSe_*x*_ is more significant in neutral media than in acidic and alkaline media, and the significant improvement of neutral HER performance of RuNC may be ascribed to the efficient transport H_2_O*/OH* in neutral media caused by RuSe_*x*_ for increasing the number of available H_2_O* on RuNC.

The importance of transport H_2_O*/OH* to neutral HER was observed via studies on kinetic isotope effect by substituting H_2_O with D_2_O in the 1.0 M KPi solution (Fig. 4f). Although the HER activities of all RuSe_*x*_–RuNC samples with different RuSe_*x*_ contents decrease significantly upon substituting H_2_O with D_2_O, the decline in performance can be partially compensated by increasing the content of RuSe_*x*_. The extracted Tafel slopes for all RuSe_*x*_–RuNC samples are in the range of 40–120 mV dec^–1^ in D_2_O solution, indicating that the initial water molecule decomposition would still proceed effectively in D_2_O (Supplementary Fig. S41). In addition, the H* and D* adsorption energies are similar in magnitude, and the effect of p*K*a can be neglected^44^. In this case, only the molecular mass of H_2_O*/OH* is changed for the neutral HER after substituting H_2_O with D_2_O, indicating that the transport of H_2_O*/OH* plays a crucial in neutral HER. Thus, the decrease of neutral HER activity in D_2_O solution is due to the decrease of the number of available H_2_O* on RuNC, and neutral RuSe_*x*_ has a positive effect on H_2_O*/OH* transport that can increase the number of H_2_O* available on RuNC remarkably.

### Operando spectroscopic analysis

In order to directly reveal how the H_2_O*/OH* transport influences the available H_2_O* in neutral media and how the available H_2_O* influences neutral HER performance at molecular level, we now discuss the interfacial water structure in acidic, neutral, and alkaline media by the operando XAS and ATR-SEIRAS (Supplementary Fig. S42).

The operando XAS data were collected at open-circuit voltage (OCV) and a representative voltage of −0.05 V (Supplementary Fig. S43). For RuSe_*x*_–RuNC, as the potential goes more negative, the Ru XANES edge shows a significant shift by 0.9 eV toward lower energy, implying a distinct decrease in the Ru valence state during neutral HER. When the applied potential returns back to the OCV (BCV), Ru XANES edge shifts back to higher energy around the initial position. In contrast, there was no significant change in the Se XANES edge as the potential decrease from OCV to −0.05 V, confirming that the HER activity comes from the Ru species rather than the Se species.

The operando ATR-SEIRAS spectra were further recorded within the HER potential range (–0.45 to 0 V). In the IR spectra, the band at ~1600 cm^−1^ is assigned to the H–O–H bending mode (*δ*_H–O–H_) of water, and the bands from 2800 to 3800 cm^−1^ are assigned to the O–H stretching mode (*ν*_O–H_) of water. An increased frequency of *ν*_O–H_ corresponds to the decreased degree of hydrogen bonds, which leads to the increased mobility of interfacial water. Specifically, the bands located near 3600, 3400, 3200, and 2900 cm^−1^ are attributed to the weak, trihedral, tetrahedral, and strong H–bonded water (i.e., free water, liquid-like water, ice-like water, and chemisorbed water), respectively^33,45^. The frequencies and/or intensities of vibrations of water exhibit a strong dependence on the electrode potential, suggesting that the obtained IR signals come mainly from the interfacial water molecules.

**Fig. 5:**
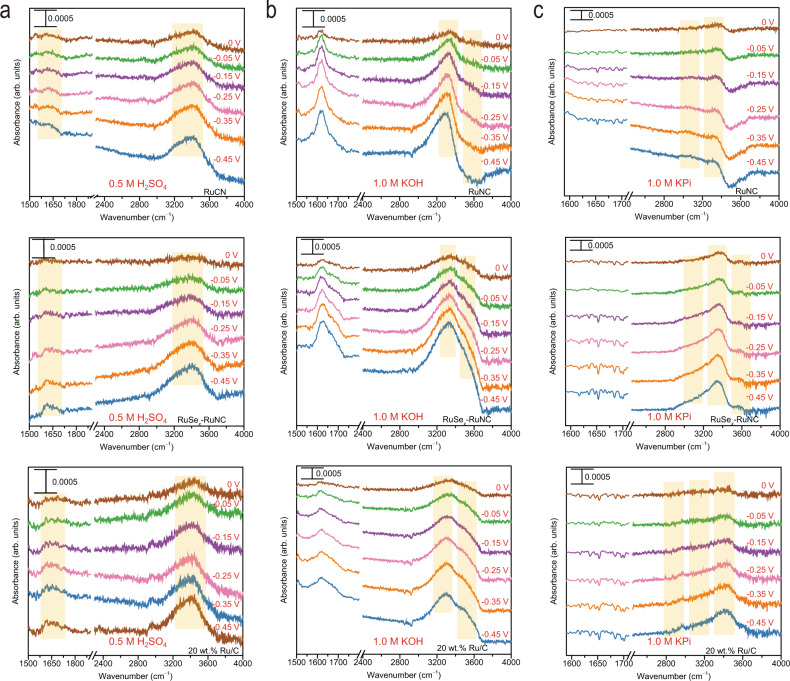
Operando spectroscopic characterizations. ATR-SEIRAS results in **a** acid, **b** alkaline, **c** neutral media, respectively.

In acid media (Fig. 5a), the *ν*_O–H_ frequencies for interfacial water are not perturbed so greatly compared with that of in neutral and alkaline media, and the liquid-like water is the unique structure of the interfacial water for all three catalysts. Shifting to more negative potentials not only results in the higher intensity of *ν*_O–H_, but also a higher intensity of *δ*_H–O–H_, indicating that the interfacial water molecule may interact with H* through the hydrogen bond of their hydrogen ends, which is in consistence with Heyrovsky step in acid media (H_3_O^+^ + e^−^ + H* → H_2_ + H_2_O). Since the H-bonded interfacial water can act as proton acceptors to facilitate the transfer of protons, the catalytic performance in acidic media is primarily affected by the adsorption of H* on the active sites in acidic media, corresponding to the similar intrinsic activities of RuSe_*x*_–RuNC and RuNC.

In contrast, the δ_H–O–H_ mode in alkaline media shows a larger Stark tuning rate than in acidic media (Fig. 5b and Supplementary Fig. S44), indicating that the interfacial water molecules in alkaline media are closer to the active sites than those of in acidic media. Notably, the liquid-like water and the free water are the unique structures of the interfacial water in alkaline media, while and shifting to more negative potentials results in more free water. In alkaline media, the non-specifically adsorbed OH^–^ anions may promote the H_2_O*/OH* to go through the interfacial water layer, generating more free water molecules to approach the active sites as proton donors to participate in the surface catalytic processes, which is consistent with the Volmer step (H_2_O + e^−^ → H* + OH^−^)^46,47^.

In neutral media (Fig. 5c), the liquid-like water and the ice-like water are the unique structures of the interfacial water, indicating that the interfacial water structure is more rigid in neutral media than in acidic and alkaline media. In addition, the absence of δ_H–O–H_ vibration bands in neutral media confirms that the interfacial water in neutral media can neither act as proton acceptors nor as proton donors. Significantly, the interfacial water structure in neutral media is very sensitive to the type of electrocatalysts. For RuNC, the band for ice–like water is more readily observed with a negative sweep of potential, and the band for free water band emerges at relatively negative voltage (~–0.15 to –0.25 V). Therefore, although RuNC can efficiently activate H_2_O*/H*, its activity in neutral media is limited by the rigid interface water, which inhibits the transport of H_2_O*/OH* at the electrode/electrolyte interface. For 20 wt.% Ru/C, although the increase in the band intensity for liquid-like water is more significant than that for ice-like water as the potential shifts negatively, the chemisorbed water exhibits a similar trend to that for liquid-like water, thereby suppressing the free water stretching. Noticeably, the bands for free and liquid-like water on RuSe_*x*_–RuNC emerge at a markedly positive voltage (~ 0 V) than those on 20 wt.% Ru/C and RuNC, providing unambiguous evidence that the disordered interfacial water network generated by RuSe_*x*_ can accelerate H_2_O*/OH* transport for increasing the number of available H_2_O* on RuNC, in consistence with our AIMD calculation results. Therefore, the interfacial water governs the rate of neutral HER, and disordering interfacial water can boost neutral HER by accelerating H_2_O*/OH* transport.

### Neutral water electrolysis device performance

**Fig. 6:**
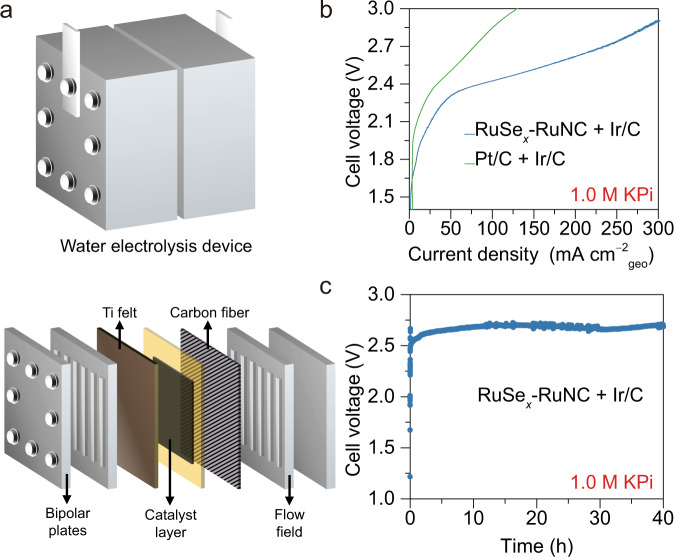
Neutral water electrolysis device performance. **a** Schematic illustration of a typical anion-exchange-membrane water electrolysis device. **b** Electrocatalytic water splitting performance of the ReSe_*x*_–RuNC + commercial Ir/C and the benchmark commercial Pt/C + commercial Ir/C measured in the neutral water electrolysis device operating at room temperature. **c** Stability tests of the ReSe_*x*_–RuNC + Ir/C-based MEA.

To highlight the practical significance of the interfacial water engineering for neutral water reduction, we further integrated RuSe_*x*_–RuNC into a membrane electrode assembly (MEA) as cathode materials with commercial Ir/C as anode materials to assemble an actual anion-exchange-membrane water electrolysis device (Fig. 6a)^48^. The current density of the MEA composed of RuSe_*x*_–RuNC + Ir/C is much higher than that of the MEA composed of benchmark commercial Pt/C + Ir/C under the same cell voltage. At a current density of 10 mA cm^–2^, the cell voltage is 1.86 V for RuSe_*x*_–RuNC + Ir/C-based MEA, which is much less than that of 2.13 V for benchmark commercial Pt/C + Ir/C-based MEA (Fig. 6b). Significantly, the RuSe_*x*_–RuNC + Ir/C-based MEA can be stably operated for at least 40 h at a larger current density of 100 mA cm^–2^ (Fig. 6c).

## Discussion

In summary, under the guidance of DFT and AIMD calculations, we designed a composite catalyst comprising Ru single atoms and RuSe_*x*_ cluster compounds; using this catalyst, we investigated the behavior of interfacial water molecules in neutral HER process. We found that, in neutral media, the interfacial water molecules are interconnected via more H-bonds than in acidic and basic media, thus inhibiting the transport of H_2_O*/OH* at the electrode/electrolyte interface. Significantly, RuSe_*x*_ can accelerate H_2_O*/OH* transport by disorder the H-bond networks at the interfacial region for increasing the number of available H_2_O* to the vicinity of RuNC, thereby boosting the activity for neutral HER. The optimized catalyst RuSe_*x*_–RuNC could deliver a current density of 10 mA cm^–2^ at an overpotential as low as 29 mV. Neutral water electrolysis device assembled with RuSe_*x*_–RuNC realizes a cell voltage of 1.86 V at a current density of 10 mA cm^–2^ and a high durability of 40 h at a larger current density of 100 mA cm^–2^. Our work here elucidates how the synergy between single atoms and cluster compounds boosts interfacial electrocatalysis at the molecular level.

## Methods

### Materials

Diethylenetriamine (C_4_H_13_N_3_, 99%), ruthenium(III) 2,4-pentanedionate (C_15_H_21_O_6_Ru, 97%), ruthenium(III) acetate (C_6_H_9_O_6_Ru, 99%), potassium hydrogen phosphate (K_2_HPO_4_, 98%), and potassium dihydrogen phosphate (KH_2_PO_4_, 99%) were purchased from Alfa. Sodium selenite (Na_2_SeO_3_, 99%) were purchased from Sigma-Aldrich. Zinc acetate dehydrate (C_4_H_10_O_6_Zn, 98%), *N*,*N*-dimethylformamide (C_3_H_7_NO, 99%), diethylamine (C_4_H_11_N, 99%), and 2-methylimidazole (C_4_H_6_N_2_, 99%) were purchased from Acros. Hydrazine hydrate (N_2_H_4_, 85%) were purchased from Sinopharm Chemical Reagent Co., Ltd. Carbon paper (TGP-H-06, the thickness is 0.19 mm) was purchased from Toray. All the above regents were used as received without any treatment. Ultrapure water (>18 MΩ) obtained from Milli-Q system was used throughout all the experiments.

### Syntheses

#### Synthesis of Ru-doped [ZnSe](DETA)_0.5_

First, carbon paper was immersed in a supersaturated C_4_H_6_O_4_Zn solution for 10 min, and then dried at 120 °C for 10 min to prepare the Zn seed covered carbon paper. Then, C_4_H_10_O_6_Zn (1.313 g), Na_2_SeO_3_ (1.037 g), C_4_H_13_N_3_ (13.5 g), N_2_H_4_ (5.2 g), and proper contents of C_15_H_21_O_6_Ru (*x*, *x* = 0, 15, 20, and 25 mg) were dissolved in H_2_O (16 g) with magnetic stirring for 10 min to form a solution. The well-dispersed solution was transferred into a 50 mL Teflon-lined stainless steel autoclave (Anhui Kemi Machinery Technology Co., Ltd) with a piece of Zn seed covered carbon paper (1.5 × 2.5 cm). The autoclave was heated at 160 °C for 20 h. After naturally cooled at room temperature, the as-prepared Ru doped [ZnSe](DETA)_0.5_/carbon paper was washed with water and absolute ethanol several times and vacuum dried at 60 °C for 12 h.

#### Synthesis of Ru-doped [ZnSe](DETA)_0.5_@Ru(acac)_3_-ZIF‐8

C_4_H_6_N_2_ (1.5 g) and proper contents of C_15_H_21_O_6_Ru (*x*, *x* = 0, 0.2, 0.3, and 0.4 mg) were dissolved in DMF (19 g) with magnetic stirring for 10 min to form a solution. The well-dispersed solution was transferred into a 50 mL centrifuge tube with a piece Ru doped [ZnSe](DETA)_0.5_/carbon paper. The mixture was heated at 80 °C for 6 h. After naturally cooled at room temperature, the as-prepared Ru doped [ZnSe](DETA)_0.5_@Ru(acac)_3_-ZIF‐8 was washed with water and absolute ethanol several times and vacuum dried at 60 °C for 12 h.

#### Synthesis of RuSe_*x*_-RuNC

A piece of Ru doped [ZnSe](DETA)_0.5_@Ru(acac)_3_-ZIF‐8/carbon paper was loaded into a ceramic boat and immersed by C_4_H_11_N, then transferred into a tube furnace with an Ar flow (50 mL min^–1^). The program of pyrolysis is: from 20 to 120 °C with a ramping rate of 1 °C min^–1^ for the evaporation of C_4_H_11_N; from 120 to 800 °C with a ramping rate of 5 °C min^–1^; kept at 800 °C for 3 h. After natural cooling, RuSe_*x*_–RuNC was obtained.

### Characterizations

Powder X-ray diffraction pattern (PXRD) was carried out with a Rigaku D/max 2500Pc X-ray powder diffractometer using Cu Kα radiation. Scanning electron microscope (SEM) was performed on a Hitachi S-4800 electron microscope. Transmission electron microscope (TEM) was carried out by a JEOL-2100F field emission microscope under 200 KV accelerating voltages. Atomic resolution high-angle annular dark-field scanning transmission electron microscopy (HAADF-STEM) were obtained by a Titan 80-300 scanning/transmission electron microscope operated at 300 kV, equipped with a probe spherical aberration corrector. Inductively coupled plasma optical emission spectrometry (ICP-OES) was carried out on a Thermo Fisher IRIS Intrepid II. X-ray photoelectron spectroscopy (XPS) was performed on a ULVAC PHI Quantera microscope. The binding energies were calibrated by C 1 s at 284.8 eV. N_2_ sorption experiments were performed at −196 °C on a Micromeritics ASAP 2420 instrument. The gas products of electrolysis were detected on a Shimazu 2010 plus gas chromatography equipped with BID and FID detectors and Shin Carbon ST 100/120 packed column. The volume of the sample loop in gas chromatograph is 1 cm^3^ and the flow rate of the gas is 20 cm^3^ min^–1^.

### Electrochemical measurements

Electrochemical performance was tested at room temperature by a Chenhua CHI 760E electrochemical workstation with a typical three-electrode setup, using a piece of as-prepared RuSe_*x*_–RuNC/carbon paper as the working electrode, a graphite rod as the counter electrode and a Ag/AgCl as the reference electrode. The H-type cell was used as the electrolyser. Nafion 117 membrane was inserted between the cathodic chamber and anodic chamber. In all, 1.0 M KPi electrolytes was prepared by diluting a mixture of K_2_HPO_4_ (107.1 g) and KH_2_PO_4_ (52.4 g) to 1 L ultrapure water. For HER tests, the electrolytes were saturated with high purity H_2_ by purging H_2_ (99.99%) into the electrolytes for 30 min. The Ag/AgCl reference electrode calibration was performed in a high-purity H_2_-saturated 0.5 M H_2_SO_4_ electrolytes using two Pt plates as both the working and counter electrode. Fast cyclic voltammetry was run at a scan rate of 50 mV s^–1^, and the average of the two potentials at which the current crossed zero was taken to be the standard potential of the Ag/AgCl reference electrode. The part of the working electrode that not contact with electrode clamp and electrolyte was coated with silica gel to prevent the capillary action, and the geometrical active area is defined by the part of the working electrode that immersed into the electrolyte (0.3 × 0.5 cm). Linear sweep voltammetry measurements were performed with a scan rate of 0.5 mV s^–1^ (all measurements were repeated for three times to ensure reproducibility). The polarization curves were corrected via 90% iR compensation: *E*_corrected_ = *E*_measured_ – 0.9 × *I*_measured_ × *R*_s_ (*E*_corrected_, *E*_measured_, *I*_measured_, and *R*_s_ represent the corrected potential, measured potential, measured current, and solution resistance, respectively). Electrochemical impedance measurements were carried with frequencies ranging from 100 kHz to 0.1 Hz at overpotential of 100 mV. Cyclic voltammetry at various scan rates (60, 80, 100, 120, 140, and 160 mV s^–1^) were collected in the non-Faradaic regions to investigate the double-layer capacitance. The electrochemical active surface area (ECSA) and the normalized current density were computed according to the equation:

$${{{{{{\rm{ECSA}}}}}}}\,=\,\frac{{C}_{{{{{{{\rm{dl}}}}}}}-{{{{{{\rm{sample}}}}}}}}}{{C}_{{{{{{{\rm{dl}}}}}}}-{{{{{{\rm{carbon}}}}}}}\;{{{{{{\rm{paper}}}}}}}}}\,\times \,S$$

$${j}_{{{{{{{\rm{ECSA}}}}}}}}\,=\,\frac{j}{{{{{{{\rm{ECSA}}}}}}}}$$

where *C*_dl_ is double-layer capacitance, *S* is the actual area of the working electrode, and *j* is current density.

### Preparation and evaluation of the neutral water electrolysis device

For a neutral water electrolysis device system, the anodic catalyst was iridium black (Johnson Matthey) for the oxygen evolution reaction (OER), and commercial Pt/C (70%, Johnson Matthey) or RuSe_*x*_–RuNC served as the cathodic catalyst for the HER. Homogeneous slurries consisting of catalysts, Nafion solution (5.0 wt.%) and isopropanol were air-sprayed onto the two sides of the anion exchange membrane with an iridium black loading of 2.0 mg cm^–2^ for the anode and 1.0 mg cm^–2^ of Pt/C and RuSe_*x*_–RuNC for the cathode. The membrane electrode assembly (MEA) was assembled into a homemade water electrolysis device with titanium mesh and carbon paper as the anodic and cathodic gas diffusion layers, respectively. In all, 1.0 M KPi was cycled on the anodic side by a peristaltic pump, and H_2_ was generated at the cathode. Polarization curves were collected from 1.0 to 3.0 V at room temperature under an ambient pressure. The stability test was carried out by galvanostatic electrolysis at a constant current density of 100 mA cm^–2^ for 40 h.

### X-ray absorption spectroscopy measurements

The XAFS spectra (Ru and Se *K*-edge) were collected at 1W1B station in Beijing Synchrotron Radiation Facility (BSRF, operated at 2.5 GeV with a maximum current of 250 mA). The data were collected in fluorescence mode using a Lytle detector while the corresponding reference sample were collected in transmission mode. The sample were grinded and uniformly daubed on the special adhesive tape. A home-made organic glass electrochemical cell was employed for the operando experiments.

### Surface-enhanced infrared absorption spectroscopy

The ATR-SEIRAS experiments were carried out by a Nicolet iS50 FT-IR spectrometer equipped with a chemically deposited ultra-thin Au film as working electrode for IR-signal enhancement^49^.

### Theoretical calculation

#### Computational models

The calculated lattice constant of Ru is 2.733 × 2.733 × 4.265 Å, and the calculated lattice constant of graphene is 2.465 × 2.465 × 7.024 Å. The Ru(001)–water interfaces, RuSe_*n*_–water interfaces, and RuN_4_–water interface were modeled by fully filling the vacuum space between the slabs with water molecules, keeping the density of water in the bulk 1 g mL^–1^. Ru(001)–water interface contains 96 Ru atoms and 72 water molecules with a size of 10.934 × 9.469 × 35.724 Å, consisting of two symmetric interfaces. RuSe_*n*_–water interface was modeled by adsorption Se atom on the *hcp* sites of the Ru (001). The RuN_4_–water interface contains 52 C atoms, 8 N atoms, 2 Ru atoms, and 72 water molecules with a size of 9.860 × 9.860 × 50.689 Å.

#### Electronic structure calculations

Electronic structure calculations were performed by plane-wave-based code Vienna ab initio simulation packages (VASP, version 5.4.4), with in density functional theory (DFT) framework^50^. The interactions between core and valence electrons were described by the projector augmented wave (PAW) pseudopotentials^51^. The generalized gradient approximation (GGA) in the scheme of proposed by Perdew, Burke, and Ernzerhof (PBE) was adopted to express the electron exchange correlation^52^. The long-range dispersion correction was incorporated within the PBE using Grimme’s D3 dispersion correction (PBE + D3)^53^. The *1s* electrons of H, *2s*, *2p* electrons of C, N, and O; *4s*, *4p* electrons of Se, *4p*, *4d*, *5s* electrons of Ru were treated as valence electrons. Spin polarization and symmetry is not included in the calculations. The cutoff energy of the plane-wave basis is 400 eV^54^. The First-order Methfessel–Paxton smearing function was employed to improve the convergence of states near the Fermi level with a smearing width of 0.2 eV. Dipole corrections were applied along the *z*-axis. Only the gamma point in reciprocal space was used. All atoms were fully relaxed in all dimensions till all residual forces have declined below 0.01 eV Å^–1^ and the convergence of energy and forces were set to 1 × 10^–5^ eV.

#### Kinetic energy barrier calculations

The kinetic energy barrier of the initial water dissociation step ($\Delta {G}_{{{{{{{\rm{H}}}}}}}-{{{{{\rm{OH}}}}}}}$) is calculated as follows:

$$\Delta {G}_{{{{{{{\rm{H}}}}}}}-{{{{{\rm{OH}}}}}}}={G}_{{{{{{{\rm{ts}}}}}}}}-{G}_{{{{{{{\rm{ini}}}}}}}}$$

where *G*_ts_ and *G*_ini_ are the free energy of the transient state and the initial state for water dissociation, respectively, determined by climbing-image nudged elastic band (CI-NEB) method implemented in VASP.

The free energy is calculated using the equation^55^:

$$G\,=\,\Delta E\,+\,{{{{{{\rm{ZPE}}}}}}}\,-\,{{{{{{\rm{TS}}}}}}}$$

where ZPE and TS are the zero-point energy and entropic contributions (*T* = 298.15 K), respectively.

### Density-functional-theory-based molecular dynamics

The time step for the DFTMD simulation is 0.5 fs and the canonical ensemble condition (NVT) was imposed by a Nose–Hoover thermostat with a target temperature of 300 K. The first 10 ps DFTMD simulations were treated as equilibrium periods, then followed by another 5 ps of production periods for data analysis.

Spin polarization, dipole corrections, and symmetry is not included in the calculations. The cutoff energy of the plane-wave basis is 300 eV. The First order Methfessel–Paxton smearing function was employed to improve the convergence of states near the Fermi level with a smearing width of 0.2 eV. The PBE-D3 method was employed to correct van der Waals interaction. Only the gamma point in reciprocal space was used.

## Supplementary information

Supplementary Information

## Data Availability

The data supporting the findings of this study are available within the article and its Supplementary Information.
