# Supplementary material for: Interfacial water engineering boosts neutral water reduction
Source: Nat Commun. 2022 Oct 21;13:6260. doi: 10.1038/s41467-022-33984-5 (PMC9587018; doi:10.1038/s41467-022-33984-5)
Supplement: Supplementary file 1 — Supplementary Information [file 41467_2022_33984_MOESM1_ESM.pdf]

Supplementary Information

Interfacial Water Engineering Boosts Neutral Water Reduction

Kaian Sun[1], Xueyan Wu[2*], Zewen Zhuang[1,3], Leyu Liu[1], Jinjie Fang[4], Lingyou Zeng[5], Junguo Ma[1], Shoujie Liu[1], Jiazhan Li[1], Ruoyun Dai[1], Xin Tan[1], Ke Yu[1], Di Liu[1], Weng-Chon Cheong[1], Aijian Huang[6], Yunqi Liu[5], Yuan Pan[5], Hai Xiao[1] & Chen Chen[1*]

[1] Engineering Research Center of Advanced Rare Earth Materials, Department of Chemistry, Tsinghua University, Beijing 100084, China.

[2] State Key Laboratory of Chemistry and Utilization of Carbon Based Energy Resources, College of Chemistry, Xinjiang University, Urumqi 830017, China.

[3] College of Materials Science and Engineering, Fuzhou University, Fuzhou, 350108, China.

[4] Beijing Advanced Innovation Center for Soft Matter Science and Engineering, Beijing University of Chemical Technology, Beijing100029, China.

[5] State Key Laboratory of Heavy Oil Processing, China University of Petroleum (East China), Qingdao 266580, China.

[6] School of Electronics Science and Engineering, Center for Public Security Technology, University of Electronic Science and Technology of China, Chengdu, 610054, China.

Hydrogen energy, Single atom, Cluster compounds, Interfacial water, Electrocatalysis

Table of Contents

1. Supplementary Figures

2. Supplementary Tables

3. Supplementary References

1. Supplementary Figures

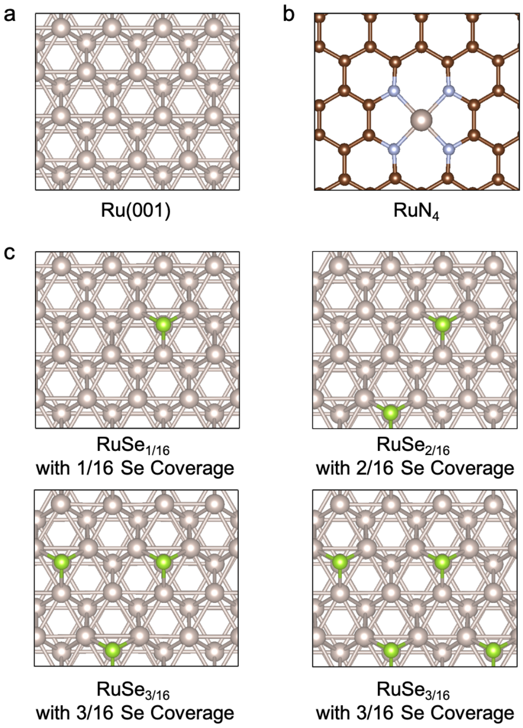

Supplementary Fig. 1 **a** Theoretical model of metal Ru(001). **b** Theoretical model of RuN_4. **c** Theoretical model of $RuSe_n$ with different Se coverage. The Ru, C, N, and Se atoms are colored with silver, brown, blue, and green, respectively.

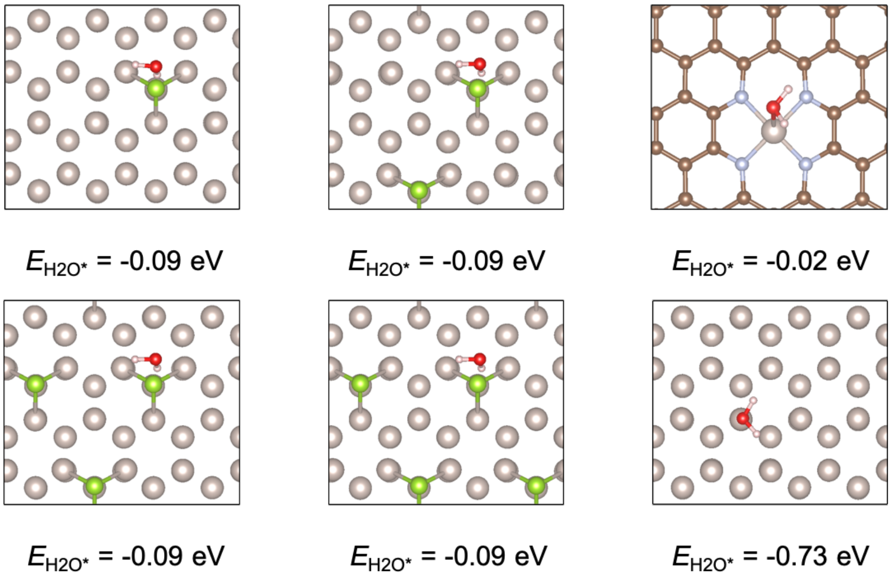

Supplementary Fig. 2 The optimized H_2O adsorption model on for $RuSe_x$, RuN_4, and Ru(001).

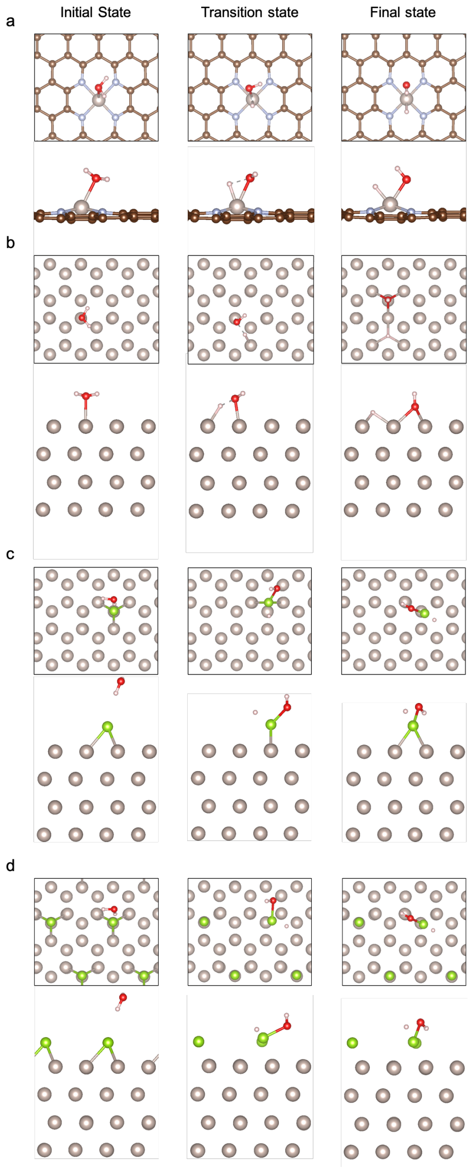

Supplementary Fig. 3 The optimized H_2O dissociation model on **a** RuN_4. **b** Ru(001). **c** $RuSe_{1/16}$-Se site. and **d** $RuSe_{1/4}$-Se site.

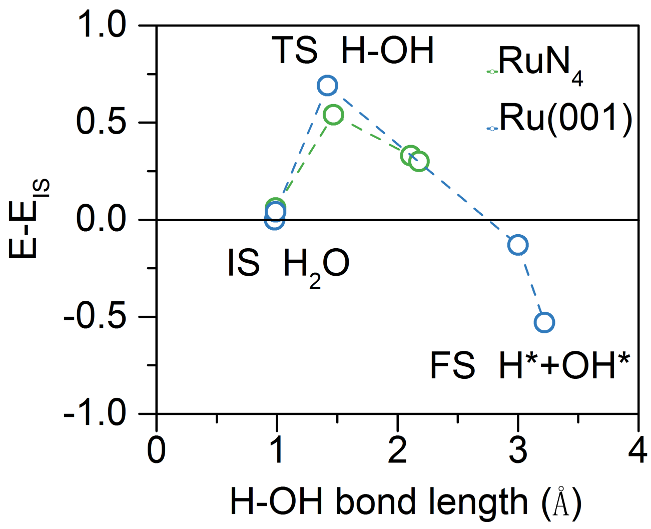

Supplementary Fig. 4 Energies along the reaction coordinate for different models. A single Ru center on RuN_4 can split water molecules, whereas the decomposition of water molecules on Ru(001) requires multi-center synergistic catalysis. This multi-center synergy leads to longer H−OH bonds in the final state of the dissociation of water molecules, which may lead to a higher decomposition energy barrier.

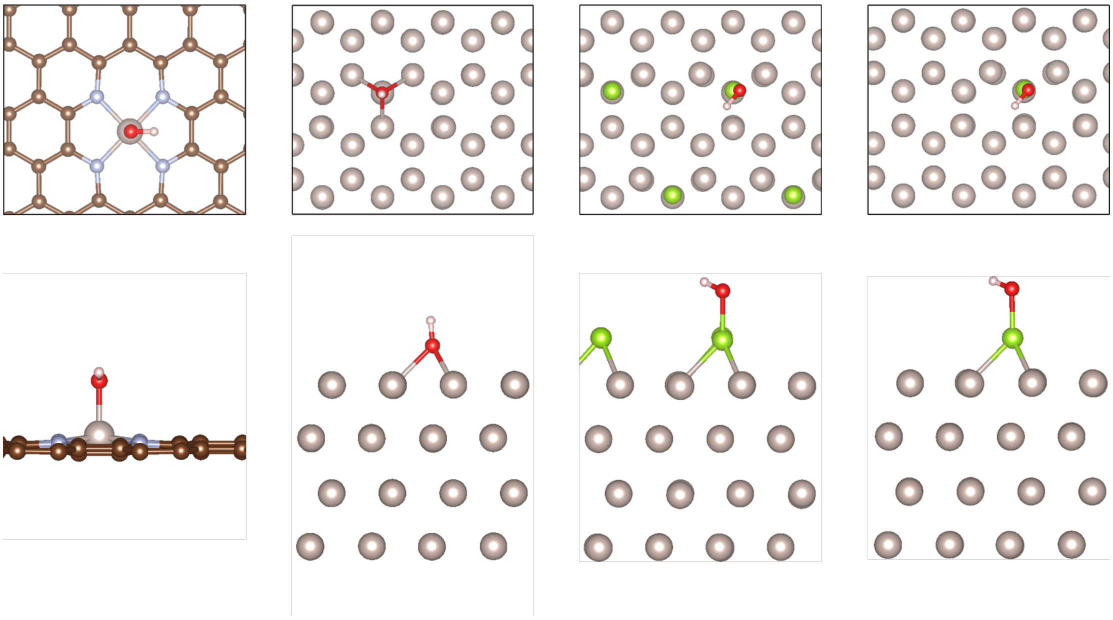

Supplementary Fig. 5 The optimized OH* adsorption model on RuN_4, Ru(001), $RuSe_{1/4}$, and $RuSe_{1/16}$ by DFT.

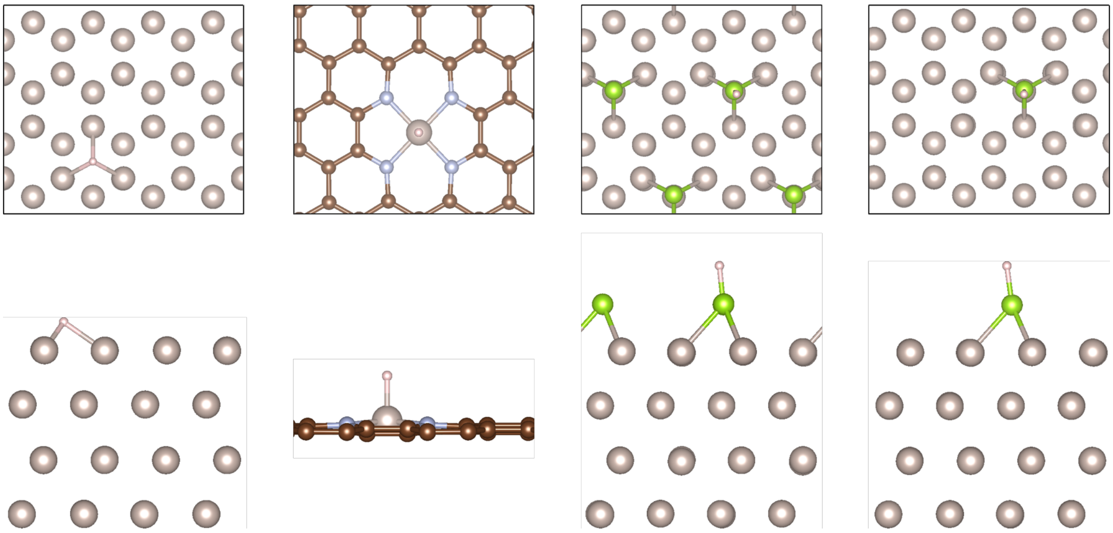

Supplementary Fig. 6 The optimized H* adsorption model on Ru(001), RuN_4, $RuSe_{1/4}$, and $RuSe_{1/16}$ by DFT.

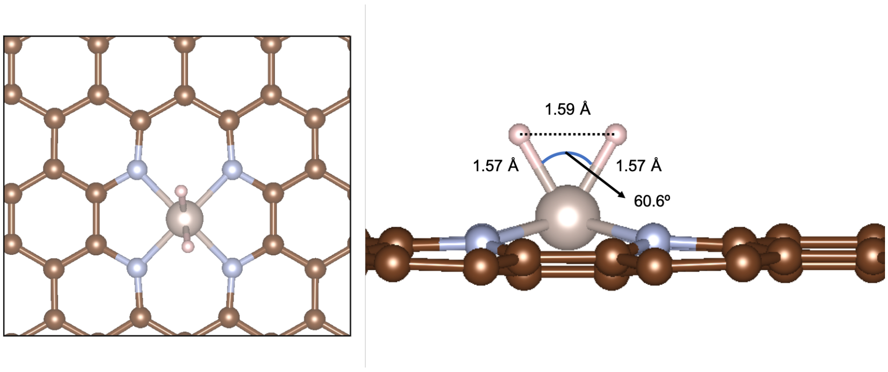

Supplementary Fig. 7 The optimized two-hydrogen adsorption model on RuN_4.

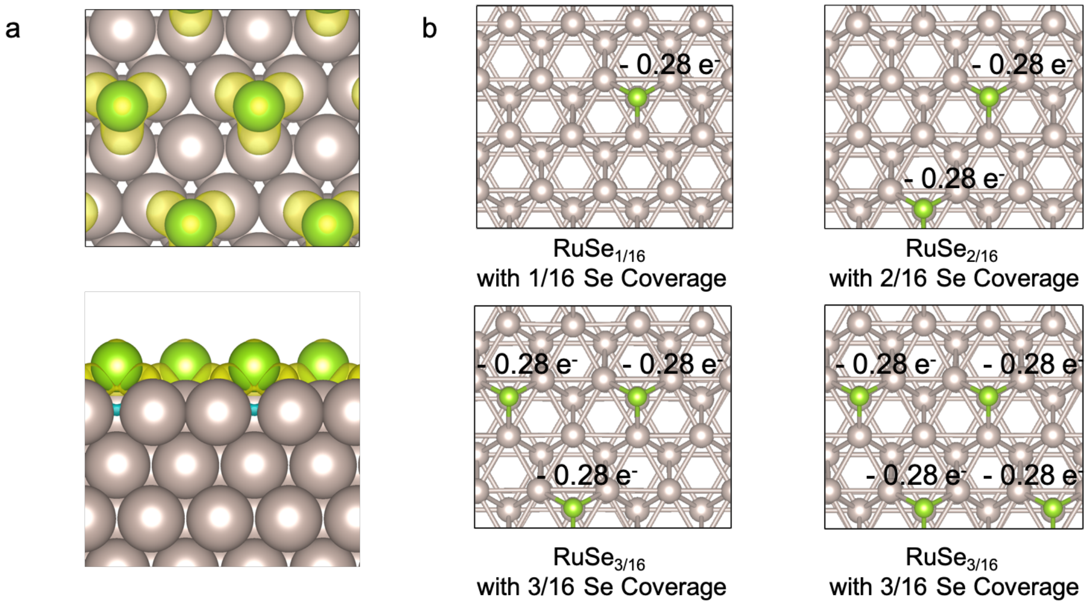

Supplementary Fig. 8 a The charge density difference for $RuSe_{1/4}$, the isosurface level is 0.003 e $Bohr^{-3}$. The yellow area represents electrons accumulation. **b** The bader charge for $RuSe_n$ with different Se coverage. The Ru atoms could act as an electron reservoir to transfer electrons to the Se atoms, so the surface electrons were relocalized on Se atoms. As the Se coverage increases from 1/16 to 1/4, the average bader charge of Se remains unchanged, so very low Se coverage can significantly localize electrons to reorient interfacial water molecules.

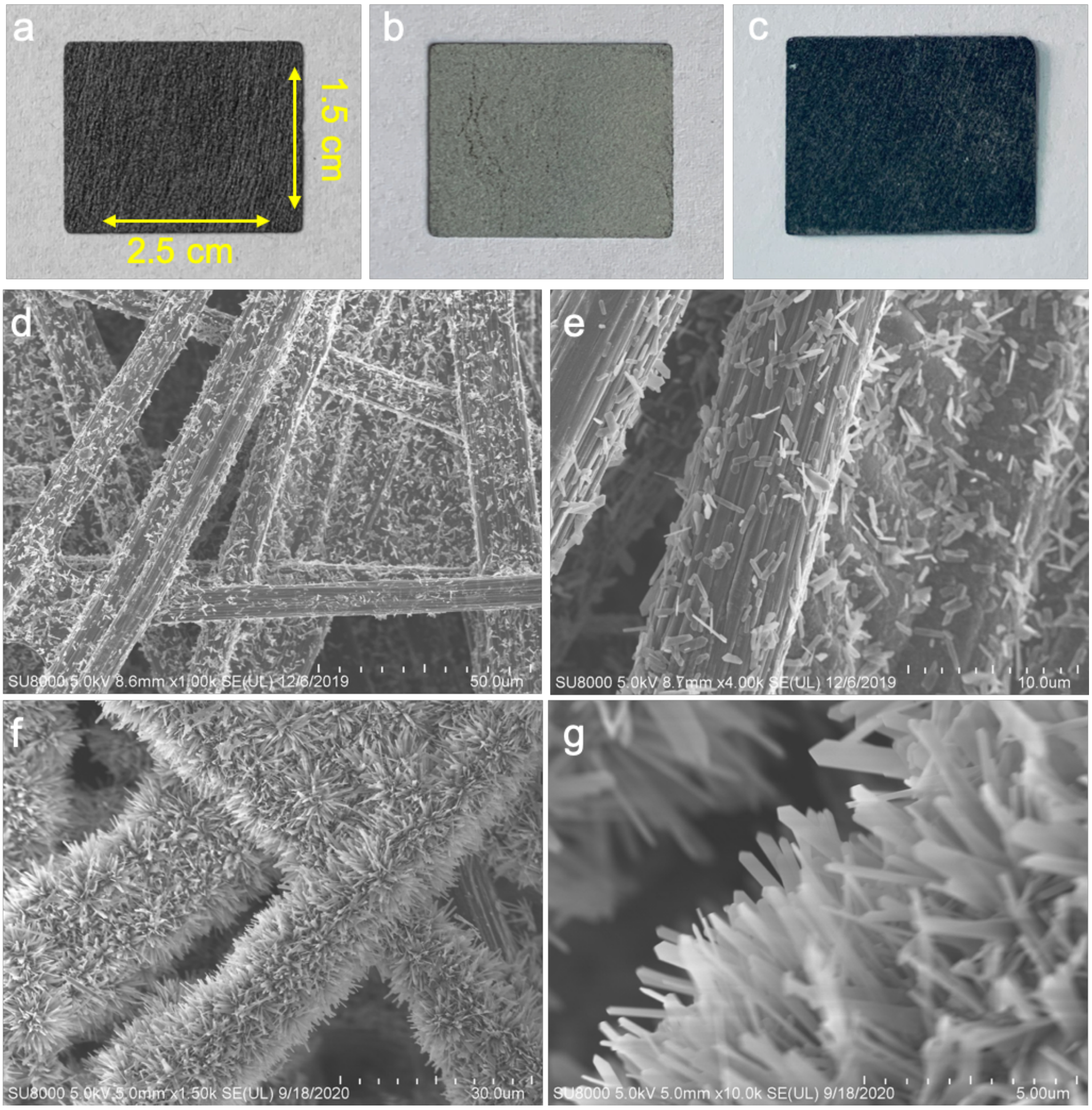

Supplementary Fig. 9 **a** The digital photograph of a pristine carbon paper. **b** The digital photograph of carbon paper with Ru-[ZnSe](DETA)$_{0.5}$ grown on one side. **c** The digital photograph of the other side of the carbon paper without Ru-[ZnSe](DETA)$_{0.5}$. **d and e** The SEM images of the Ru-[ZnSe](DETA)$_{0.5}$ grown on carbon paper without using the seed method. **f and g** The SEM image of the digital photograph of Ru-[ZnSe](DETA)$_{0.5}$ grown on carbon paper using seed method. It can be observed that Ru-[ZnSe](DETA)$_{0.5}$ was uniformly grown on carbon paper by the seed method.

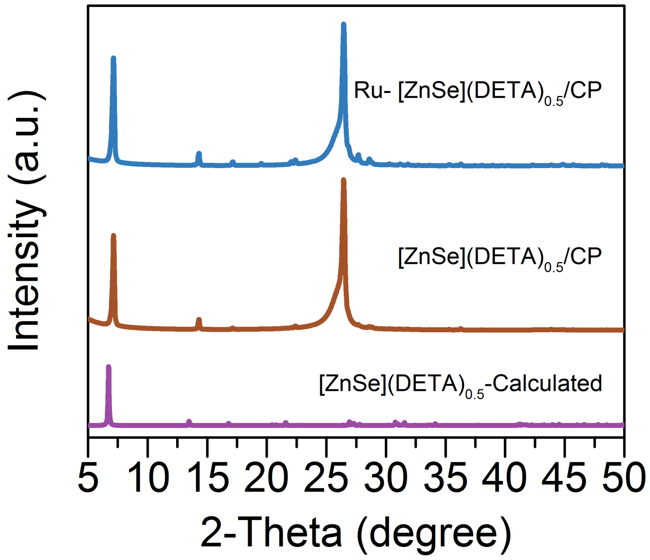

Supplementary Fig. 10 The XRD patterns of $[ZnSe](DETA)_{0.5}$ and Ru-$[ZnSe](DETA)_{0.5}$. The XRD patterns show that after introduction of Ru species, the diffraction peak of $[ZnSe](DETA)_{0.5}$ shows no significant shift.

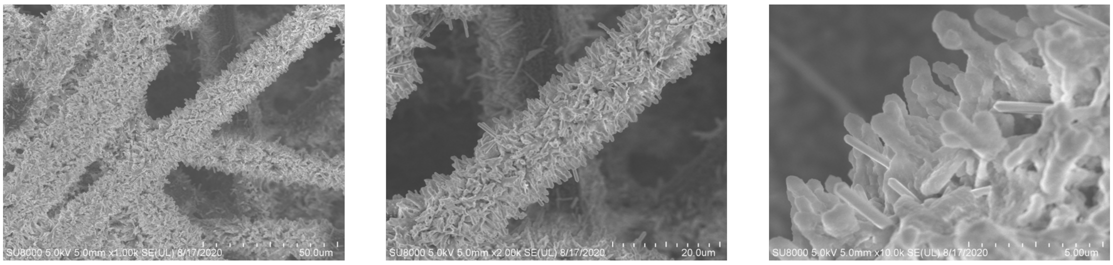

Supplementary Fig. 11 The SEM image of Ru-[ZnSe](DETA)$_{0.5}$@ZIF-8. The SEM images show that the pristine nanorod morphology of [ZnSe](DETA)$_{0.5}$ can well be retained after conversion to [ZnSe](DETA)$_{0.5}$@ZIF-8. The [ZnSe](DETA)$_{0.5}$@ZIF-8 nanorods have a diameter of 20–100 nm and lengths of 300–500 nm, in which ZIF-8 layer with irregular surface encapsulates the nanorods completely.

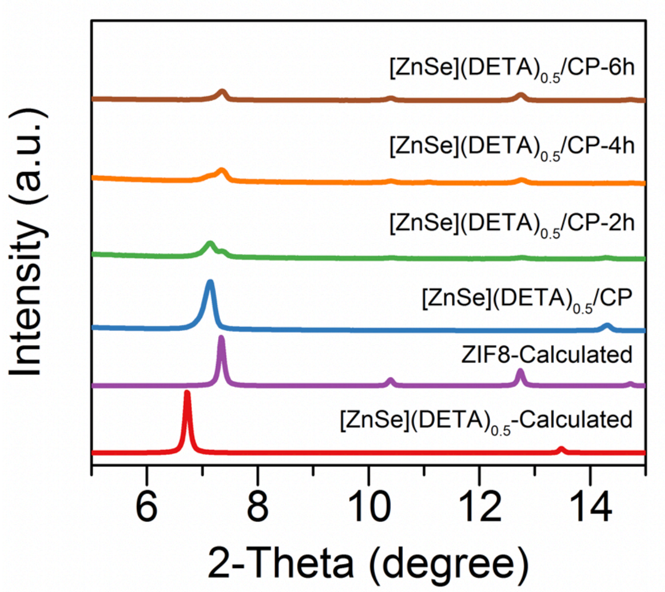

Supplementary Fig. 12 The XRD patterns of $[ZnSe](DETA)_{0.5}$@ZIF-8 prepared by different reaction time. The XRD patterns show that as the reaction time increases, the characteristic diffraction peaks of $[ZnSe](DETA)_{0.5}$ gradually weaken, and the diffraction peak of ZIF-8 gradually increased. After reaction for 6 h, the diffraction peak of $[ZnSe](DETA)_{0.5}$ disappeared completely, indicating that $[ZnSe](DETA)_{0.5}$ was completely transformed into ZIF-8.

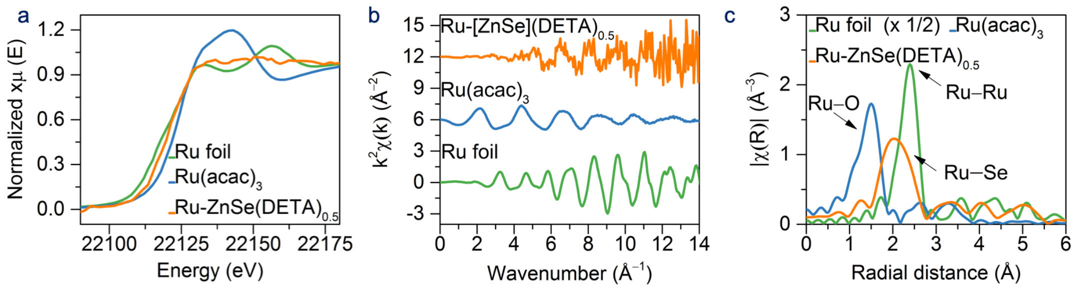

Supplementary Fig. 13 a The Ru K-edge XANES spectra. **b** The Ru K-edge extended XAFS oscillation functions $k^2\chi(k)$. **c** The corresponding k^2-weighted Fourier Transform spectra of Ru K-edge extended XAFS. It can be observed that, in comparison with Ru(acac)$_3$ and Ru foil, Ru-[ZnSe](DETA)$_{0.5}$@ZIF-8 shows an addition peak at 2.0 Å, which can be ascribed to the presence of Ru–Se nearest-neighboring coordination, confirming the formation of Ru…Se species.

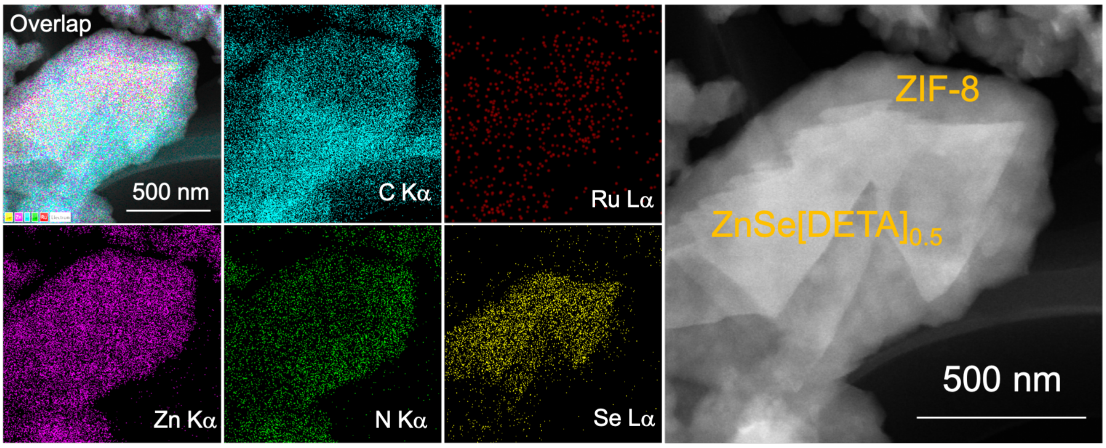

Supplementary Fig. 14 The HAADF-STEM and EDS mapping images of Ru-[ZnSe](DETA)$_{0.5}$@ZIF-8 after reaction for 2 h. The HAADF-STEM and EDS mapping images show that the conversion from [ZnSe](DETA)$_{0.5}$ to ZIF-8 starts from the edge of [ZnSe](DETA)$_{0.5}$ and the Ru…Se species are uniformly dispersed in ZIF-8.

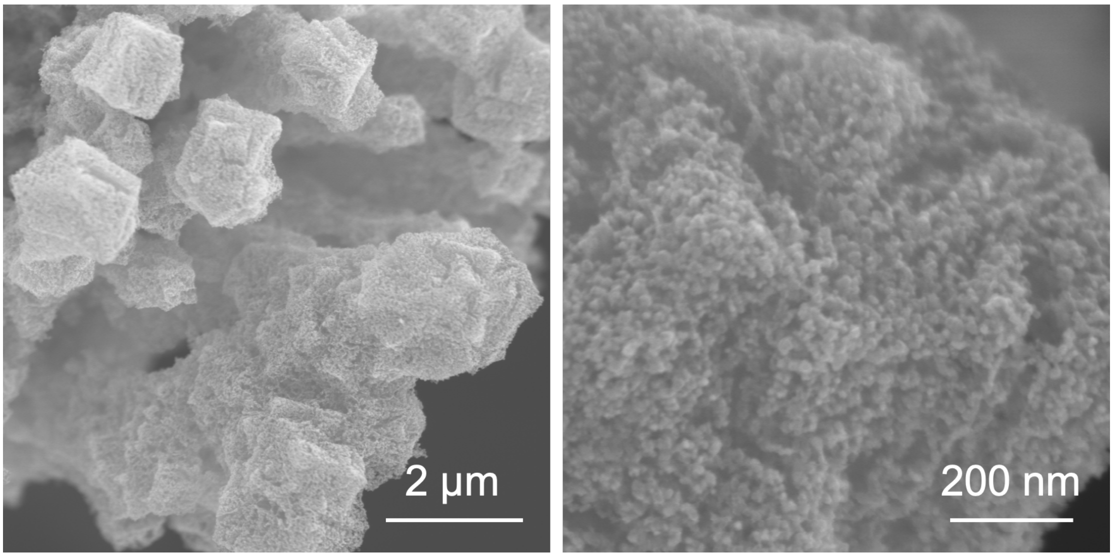

Supplementary Fig. 15 The SEM images of $ReSe_x$–RuNC.

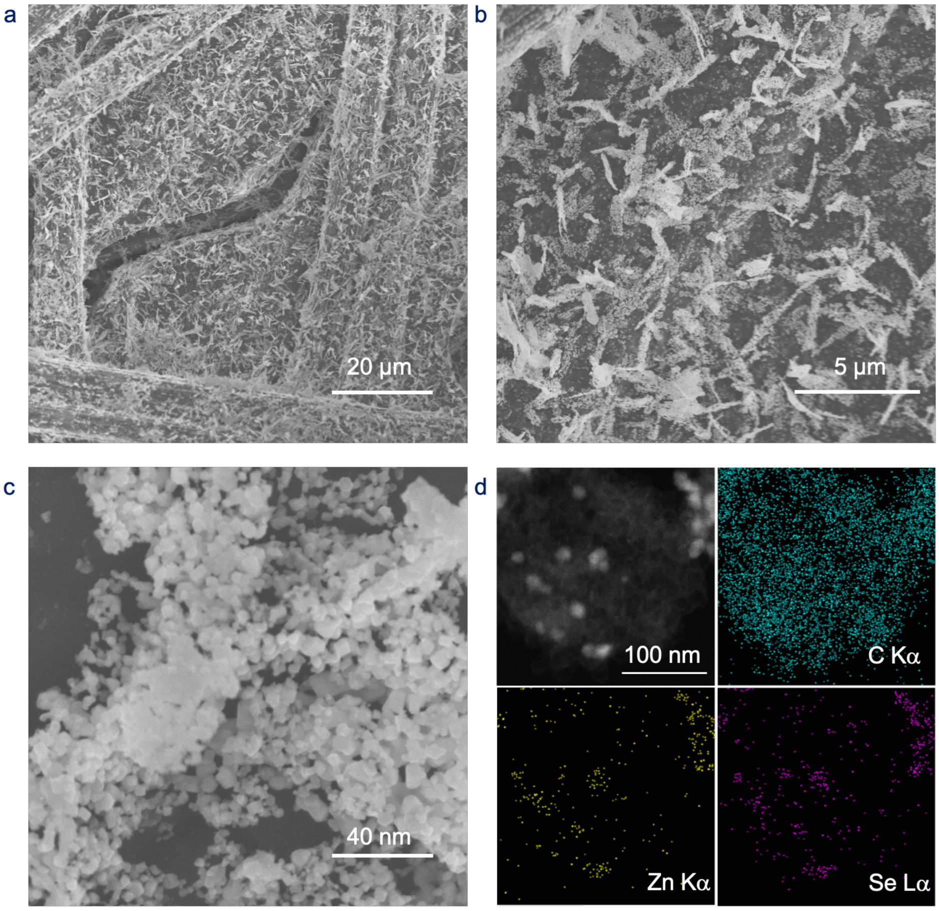

Supplementary Fig. 16 a-c The SEM images of Ru-[ZnSe](DETA)$_{0.5}$@Ru(acac)$_3$-ZIF-8 pyrolyzed at 750 °C under air atmosphere. **d** The HAADF-STEM and EDS mapping images of Ru-[ZnSe](DETA)$_{0.5}$@Ru(acac)$_3$-ZIF-8 pyrolyzed at 750 °C under Ar atmosphere. The SEM images show that the nanorods morphology remains after calcination under air conditions. Notably, the C elements were evaporated during calcination process under air conditions, and the nanorods consisted of ZnSe$_x$ nanoparticles with a size of about 10–30 nm, similar to the size of hollow carbon spheres. In contrast, EDS mapping further indicated that during the calcination process under Ar conditions, with the increase of calcination temperature, ZnSe$_x$ nanoparticles were gradually evaporated, and the remaining C elements gradually graphitized into a hollow spherical network.

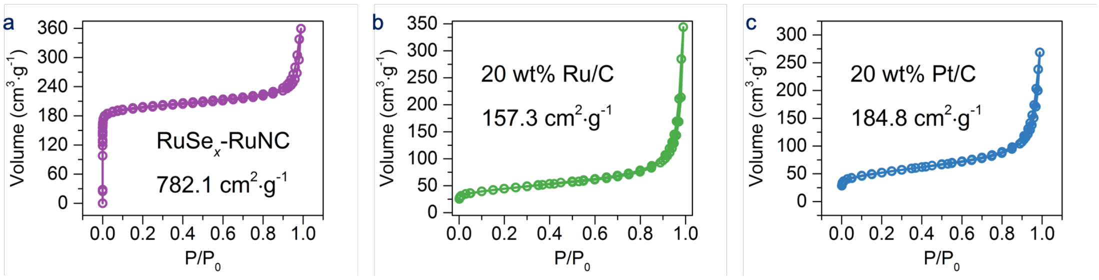

Supplementary Fig. 17 The N_2 adsorption-desorption isotherms of **a** RuSe$_x$–RuNC. **b** commercial Ru/C. **c** commercial Pt/C catalyst.

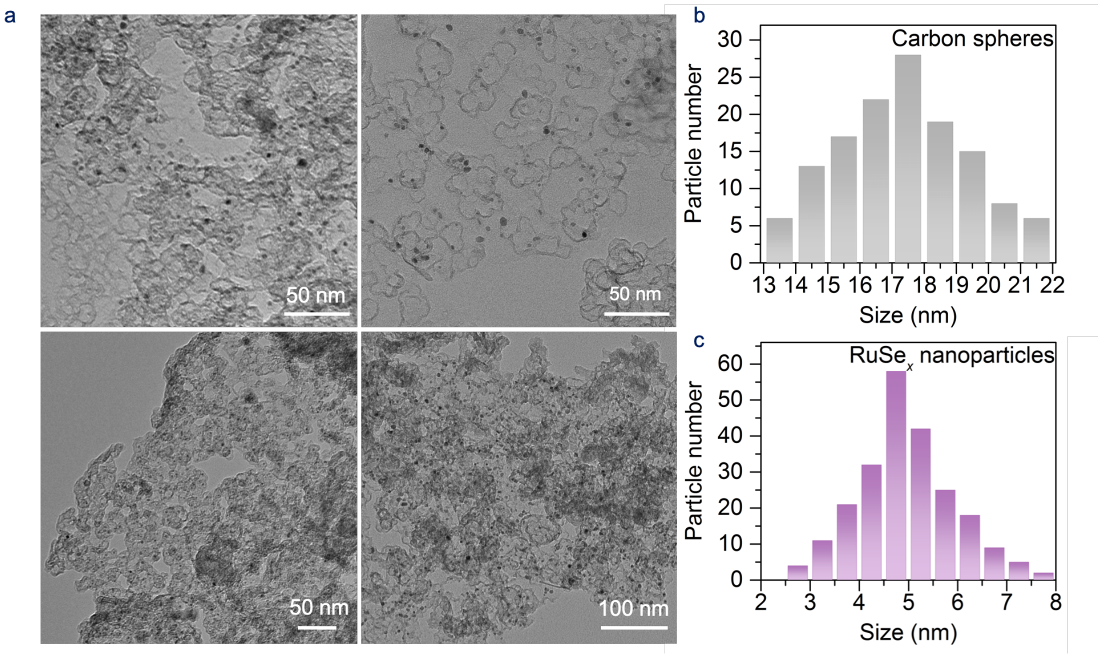

Supplementary Fig. 18 a The TEM images of RuSe$_x$ NPs within spherical carbon network. **b** The corresponding size distribution of carbon spheres. **c** The corresponding size distribution of RuSe$_x$ NPs.

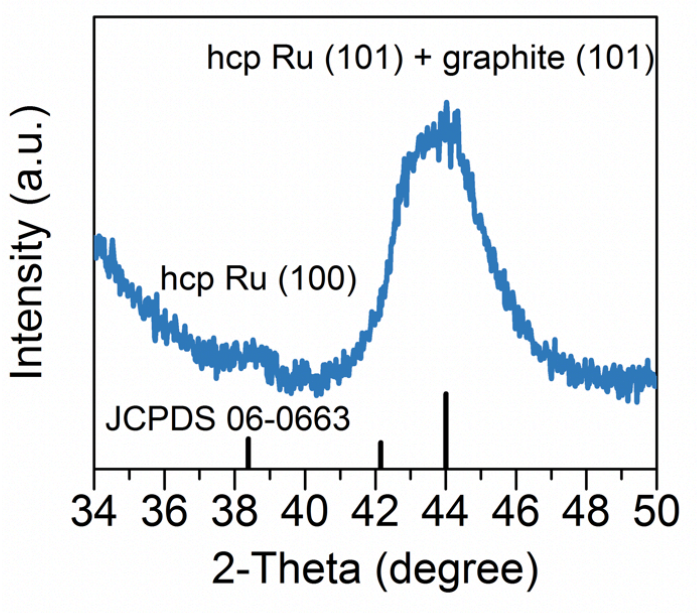

Supplementary Fig. 19 The XRD patterns of $RuSe_x$.

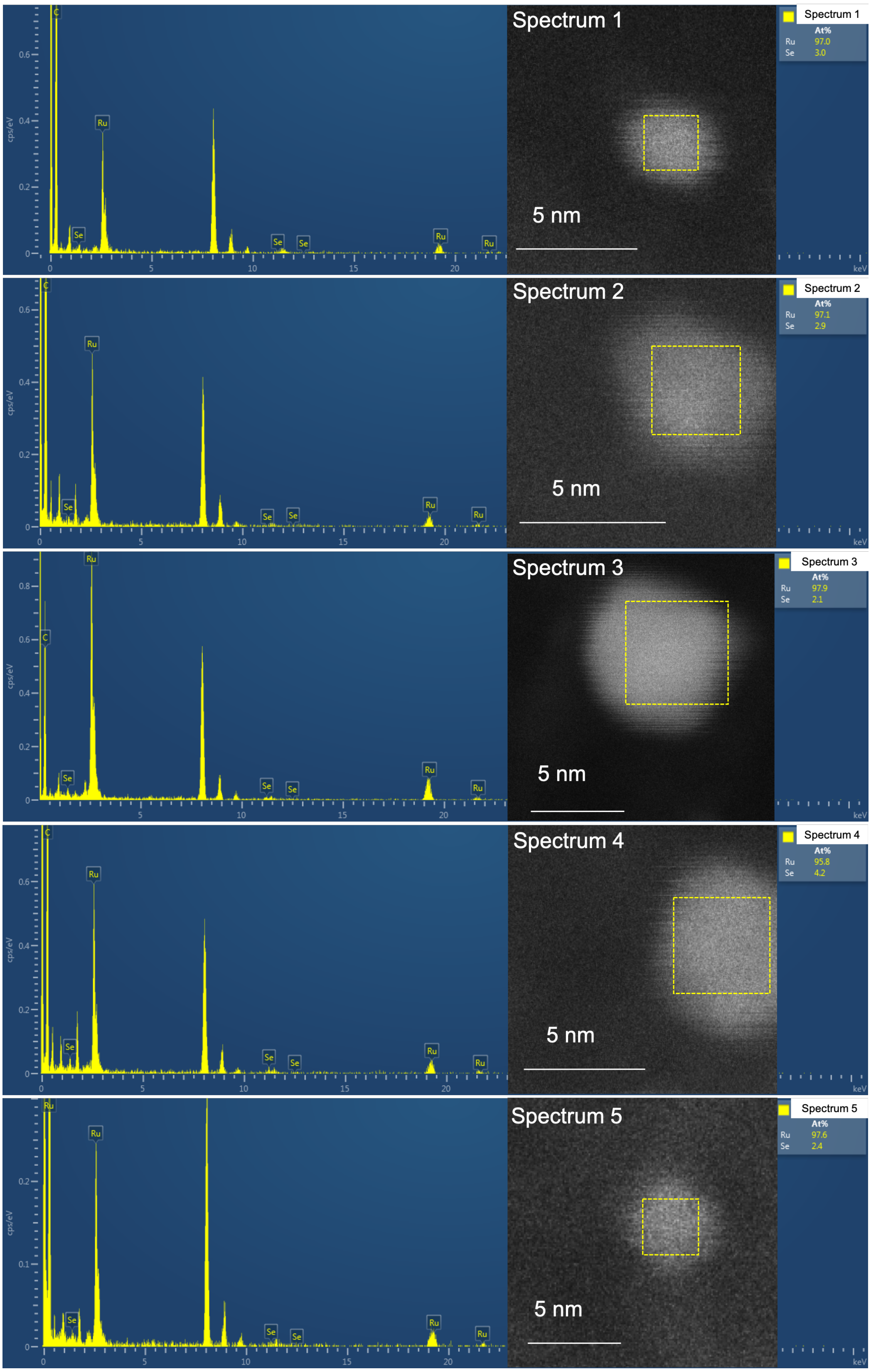

Supplementary Fig. 20 The EDX analysis under HAADF-STEM mode.

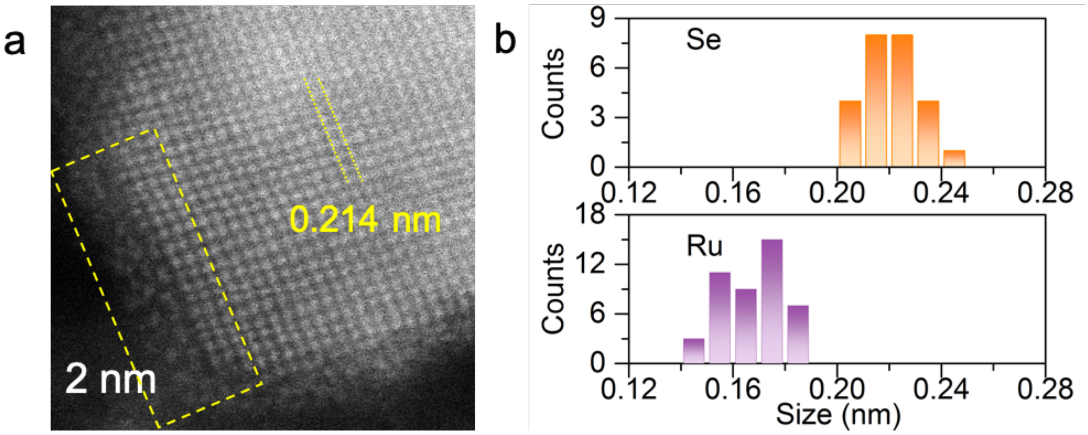

Supplementary Fig. 21 **a** The sub-Å resolution HAADF-STEM images of $RuSe_x$. **b** The corresponding size distribution of Ru and Se atoms. As revealed by the sub-Å resolution HAADF-STEM image, the Ru core features a single-crystalline structure oriented along the [002] direction. A number of Se atom columns (in dark contrast) are on the outermost surface of brighter Ru atom columns, which results from smaller atomic number of the former than the latter. Since the atomic radius of Se (2.55 Å) is larger than that of Ru (1.89 Å), the Se atom columns (with diameters of ~2.21 Å) show an atomic stacking mode different from the underlying Ru atom columns (with diameters of ~1.68 Å), providing direct evidence for the existence of an amorphous Se cluster with low coverage without epitaxial growth to form lattice fringes.

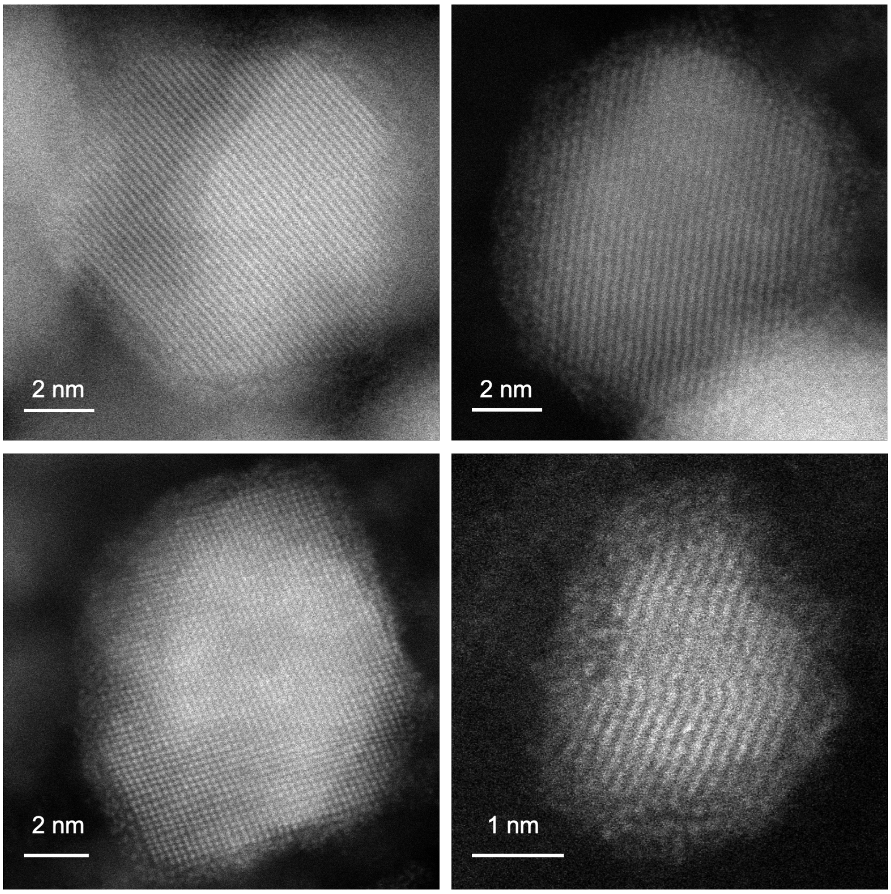

Supplementary Fig. 22 The sub-Å resolution HAADF-STEM images of $RuSe_x$ nanoparticles. The sub-Å resolution HAADF-STEM images confirm that the Ru cores in $RuSe_x$–RuNC are uniformly stabilized by amorphous Se atoms.

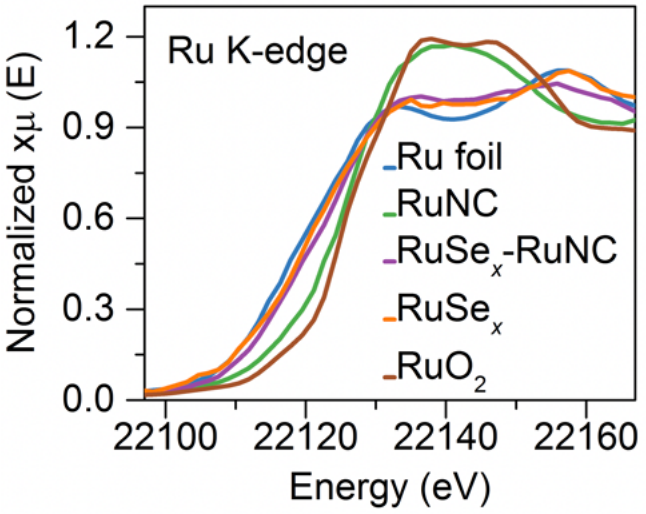

Supplementary Fig. 23 The Ru K-edge XANES spectra.

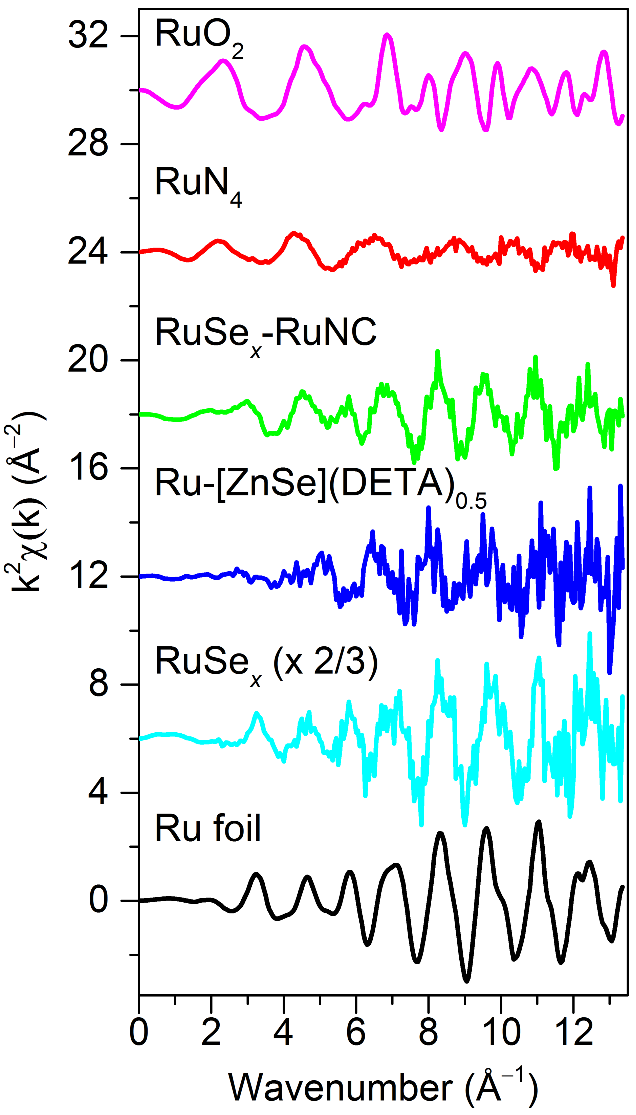

Supplementary Fig. 24 The Ru K-edge extended XAFS oscillation functions $k^2\chi(k)$.

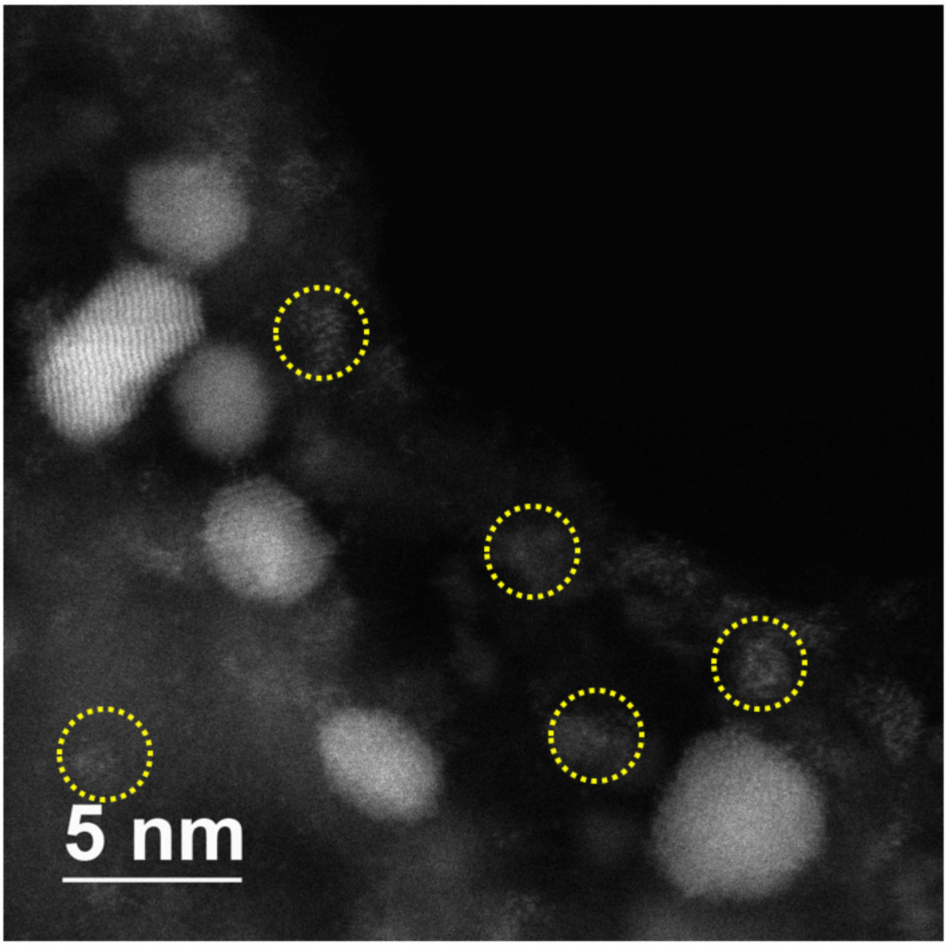

Supplementary Fig. 25 The sub-Å resolution HAADF-STEM images of $RuSe_x$–RuNP.

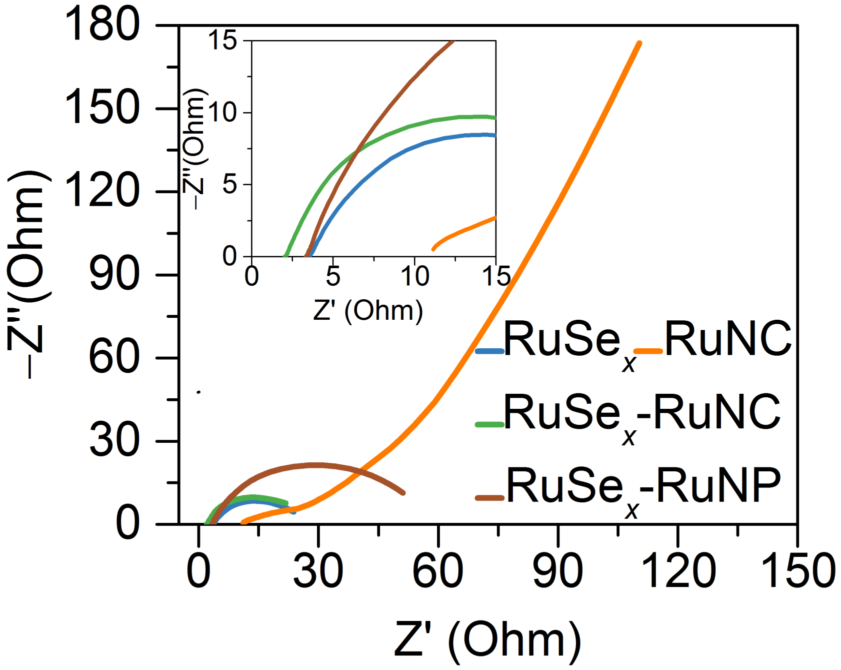

Supplementary Fig. 26 Electrochemical impedance spectra. The R_s values for RuSe$_x$–RuNC, RuSe$_x$–RuNP, RuSe$_x$, and RuNC are 2.1, 3.2, 3.5, and 11.1 Ω, respectively.

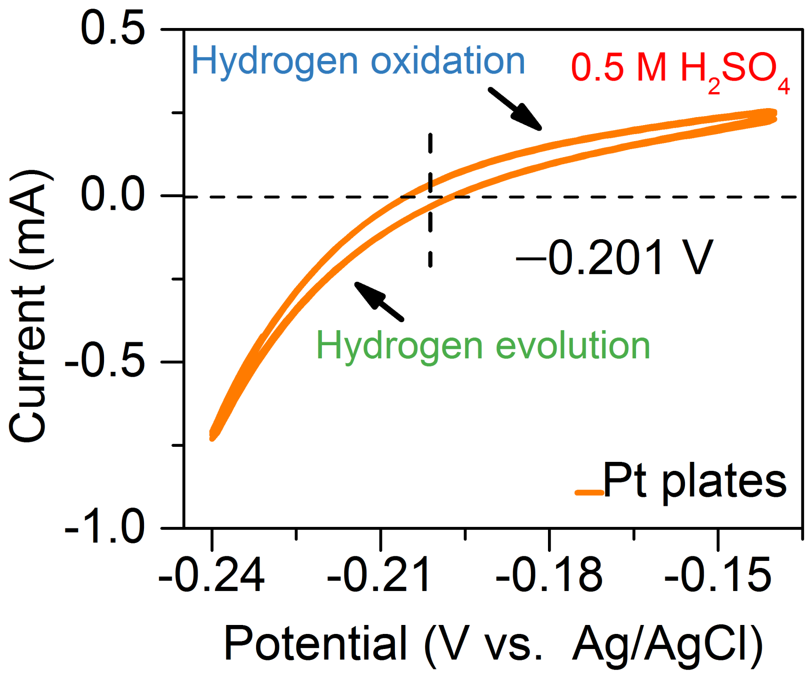

Supplementary Fig. 27 The Ag/AgCl reference electrode calibration. The calibrated E(Ag/AgCl) value is –0.201 V.

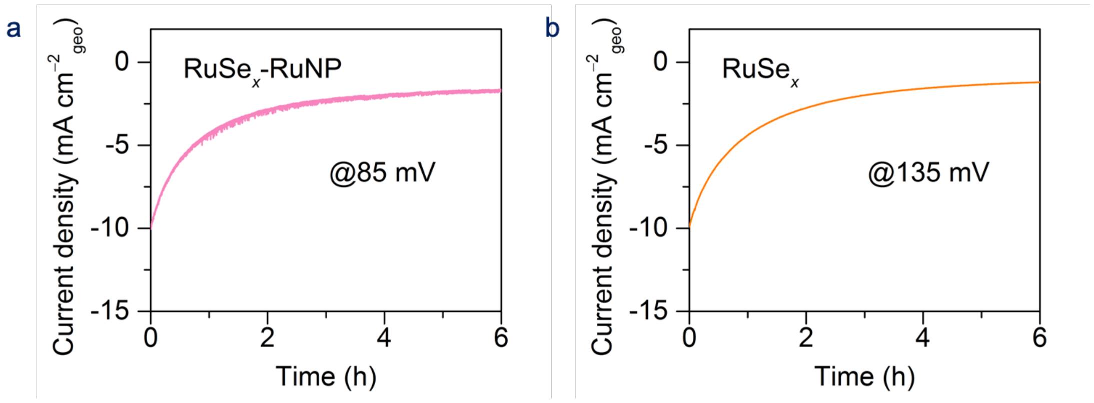

Supplementary Fig. 28 The chronopotentiometric curves of **a** RuSe$_x$–RuNP. **b** RuSe$_x$.

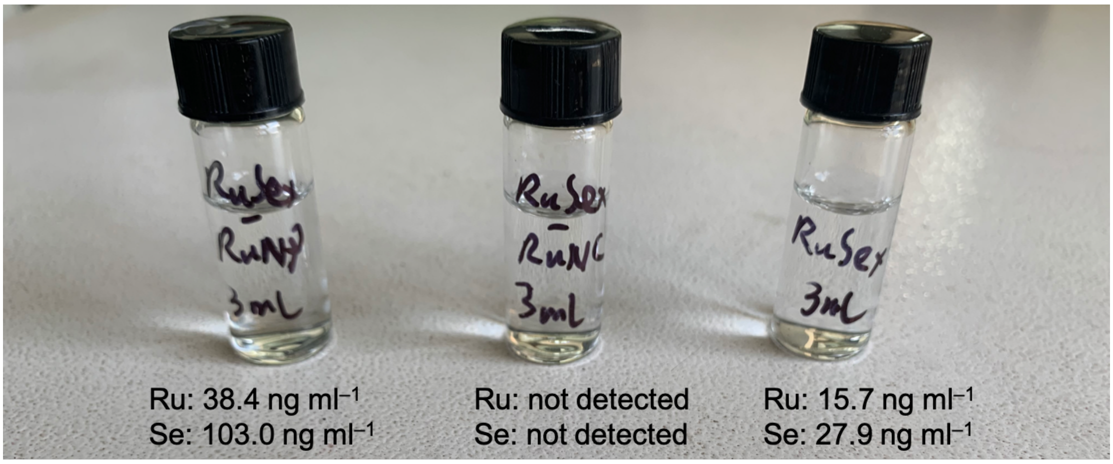

Supplementary Fig. 29 The Ru and Se content determined by the ICP-OES analysis of the electrolytes after 6 h chronopotentiometric tests. Without the assistance of RuNC, the Ru species in $RuSe_x$ and RuSex–RuNP were obviously lost. The dissolution of Ru species on cathodes composed of Ru-based bulk materials has been reported in previous articles[1,2], which is probably the main reason for the deactivation of $RuSe_x$ and $RuSe_x$–RuNP. In contrast, since the active center in $RuSe_x$–RuNC is RuNC, which is efficient and stable for HER, the loss of Ru species could be greatly suppressed.

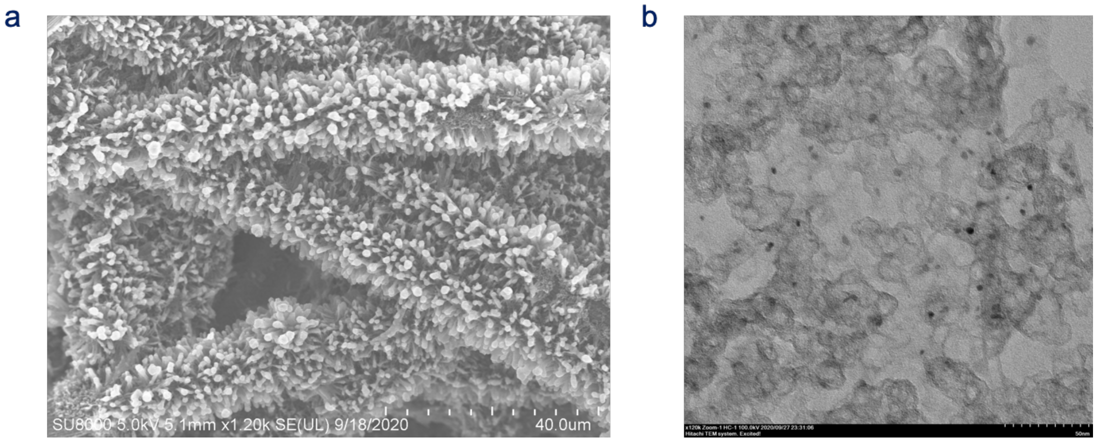

Supplementary Fig. 30 a The SEM image of used $RuSe_x$–RuNC. **b** The TEM image of used $RuSe_x$–RuNC.

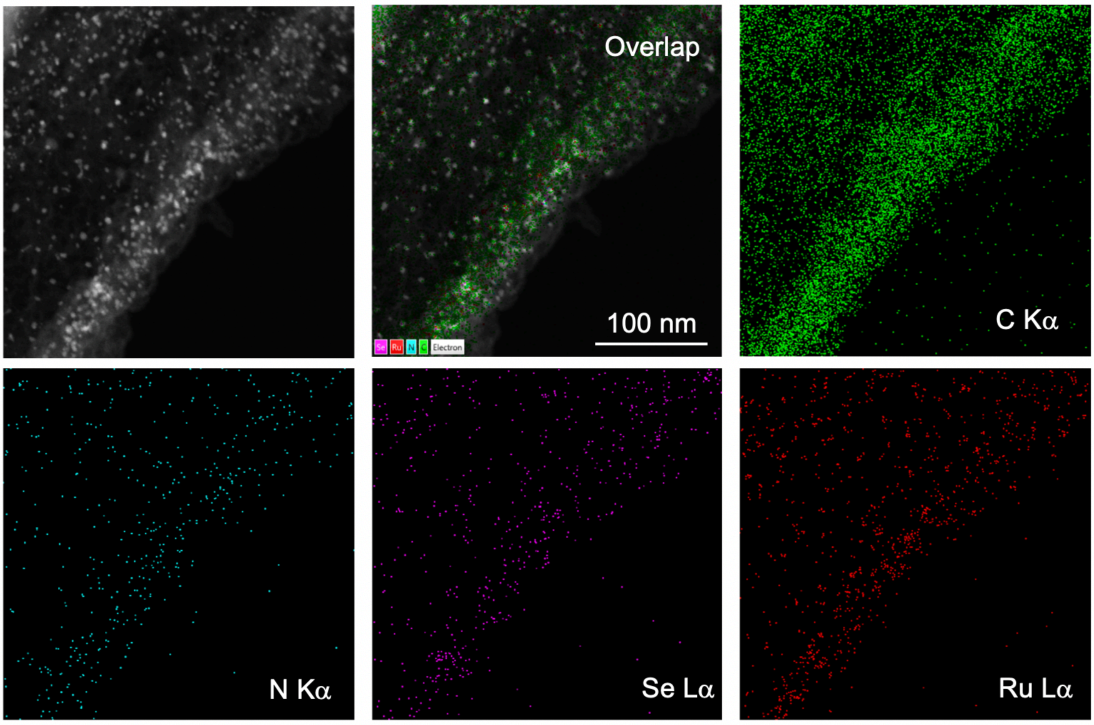

Supplementary Fig. 31 The HAADF-STEM and EDS mapping images of used $RuSe_x$–RuNC.

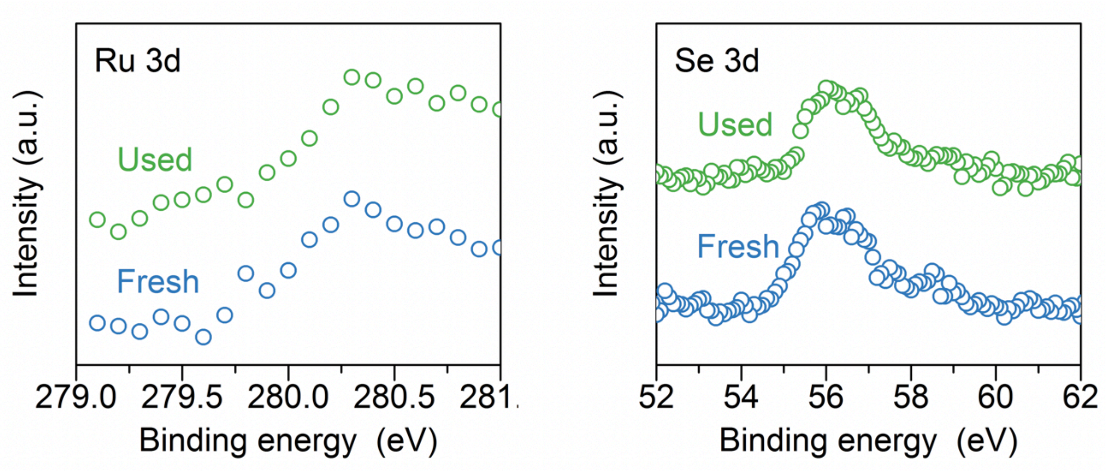

Supplementary Fig. 32 The XPS spectra of fresh and used $RuSe_x$–RuNC.

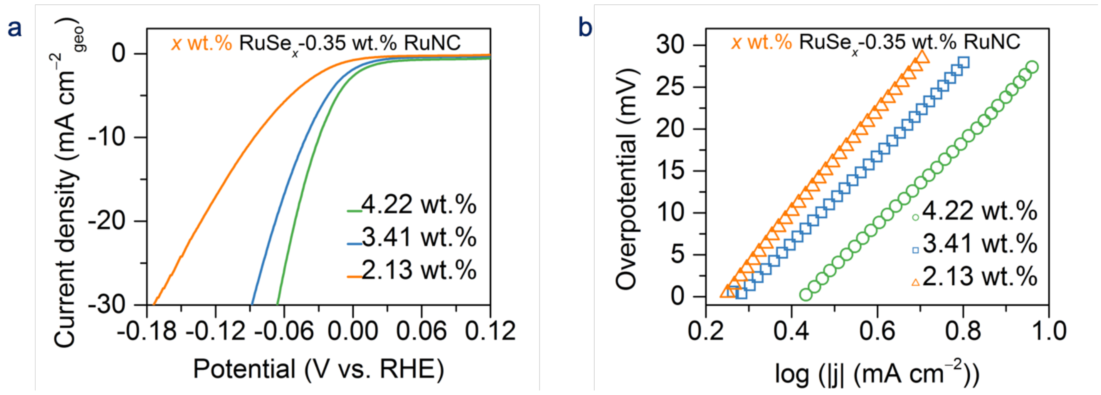

Supplementary Fig. 33 **a** The LSV curves of RuSe$_x$–RuNC with different RuSe$_x$ contents. **b** The corresponding Tafel slopes.

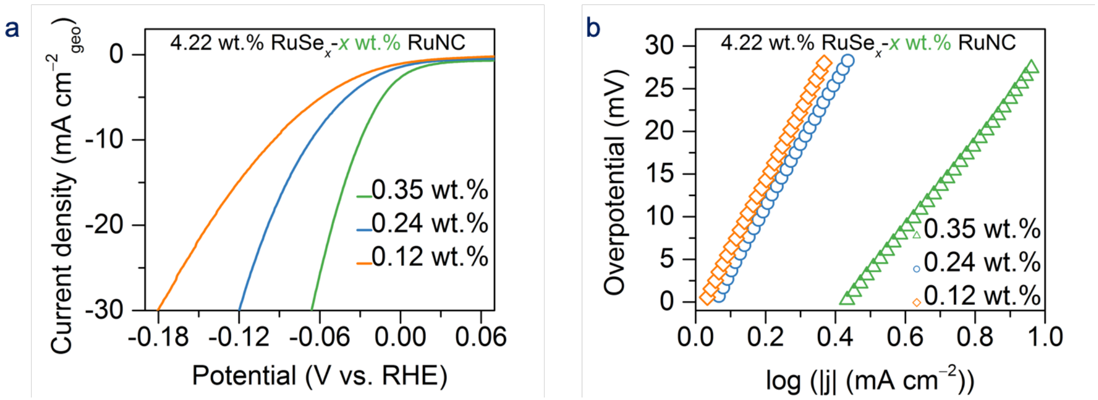

Supplementary Fig. 34 a The LSV curves of $RuSe_x$–RuNC with different RuNC contents. **b** The corresponding Tafel slopes.

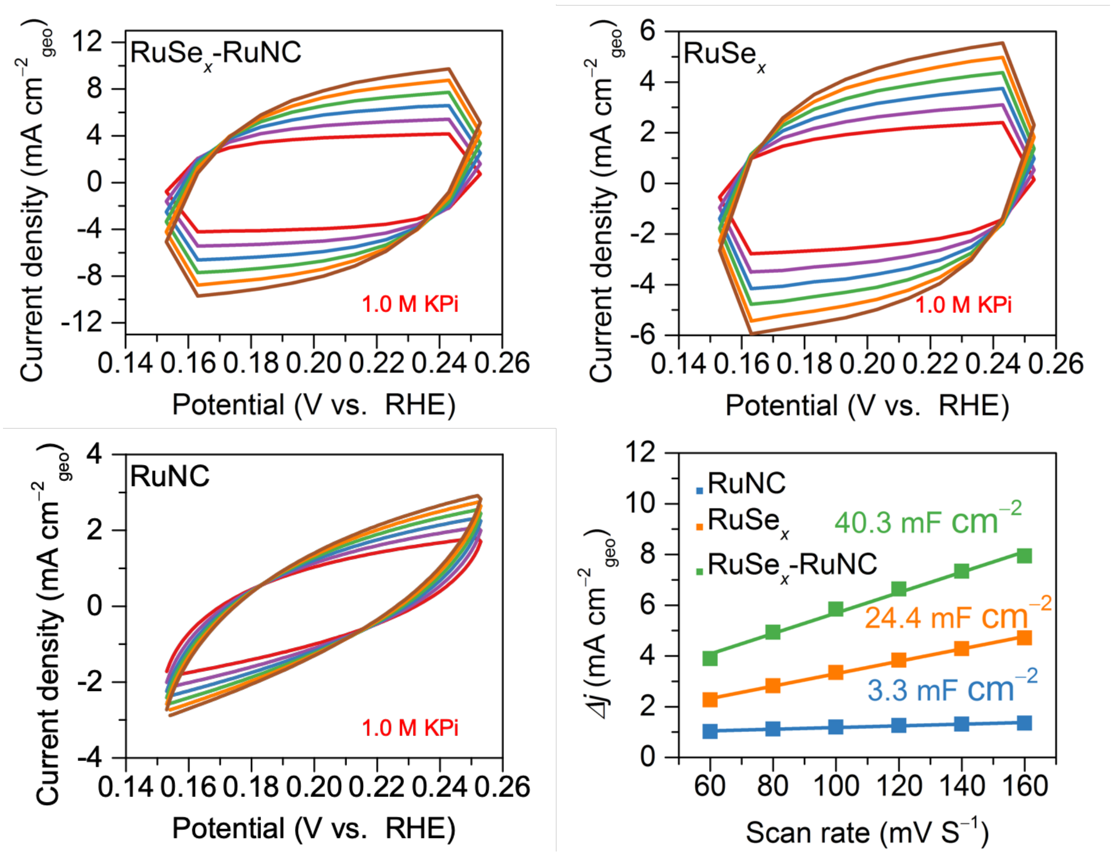

Supplementary Fig. 35 Cyclic voltammetry measured from 60 to 160 mV s^{-1} of $RuSe_x$-RuNC, $RuSe_x$, and RuNC under neutral media and the corresponding Δj vs. scan rates plots.

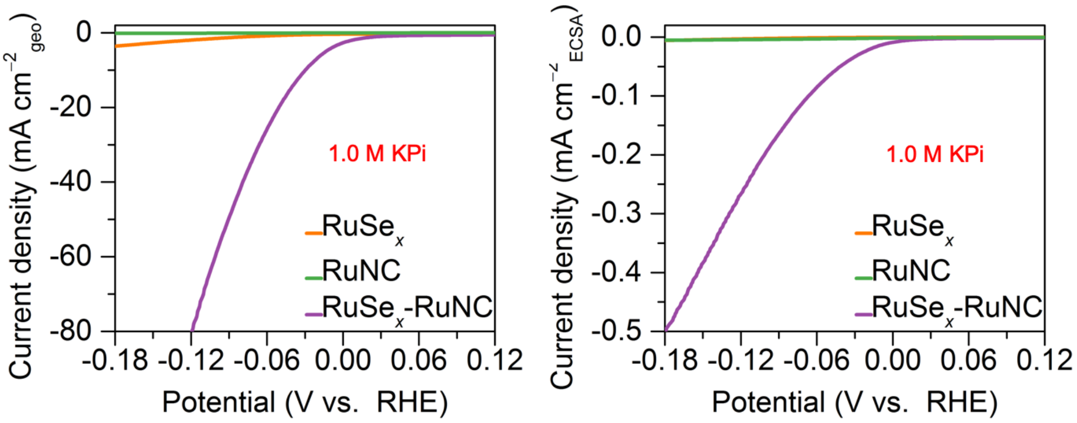

Supplementary Fig. 36 The linear sweep voltammetry curve per geometric area and the linear sweep voltammetry curve per ECSA in neutral media.

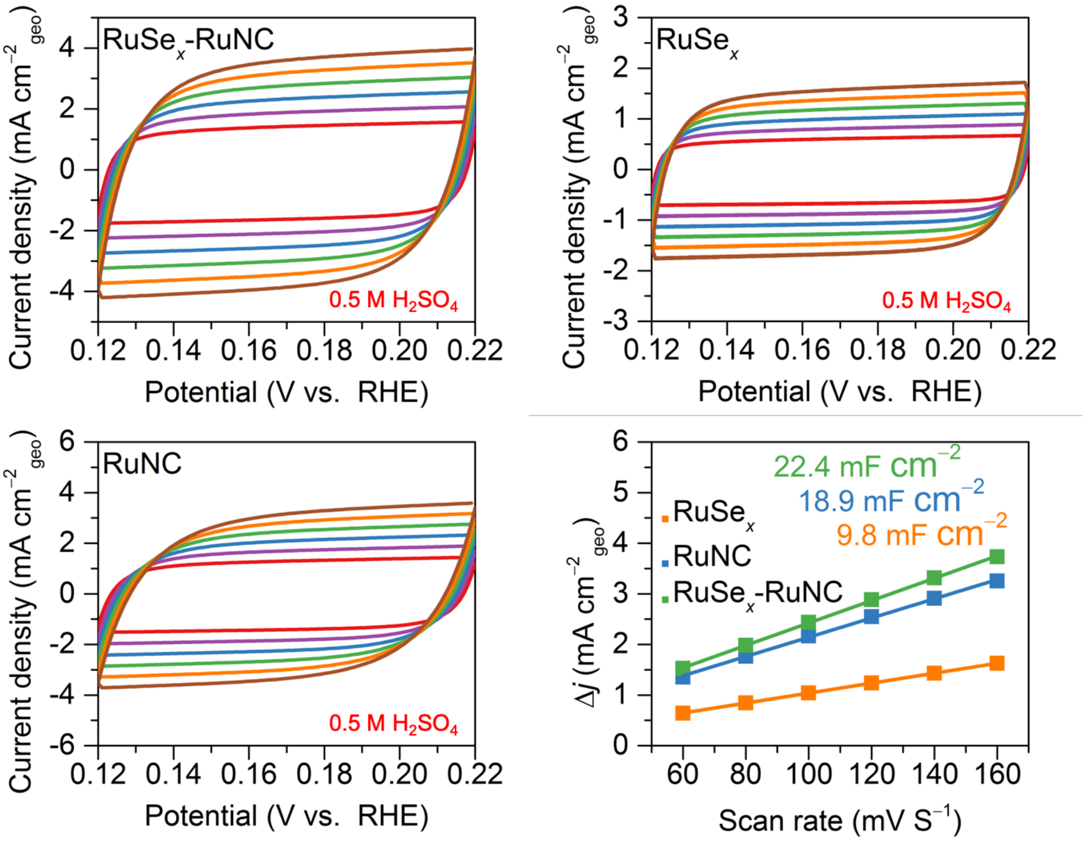

Supplementary Fig. 37 Cyclic voltammetry measured from 60 to 160 mV s^{-1} of $RuSe_x$-RuNC, $RuSe_x$, and RuNC under acidic media and the corresponding Δj vs. scan rates plots.

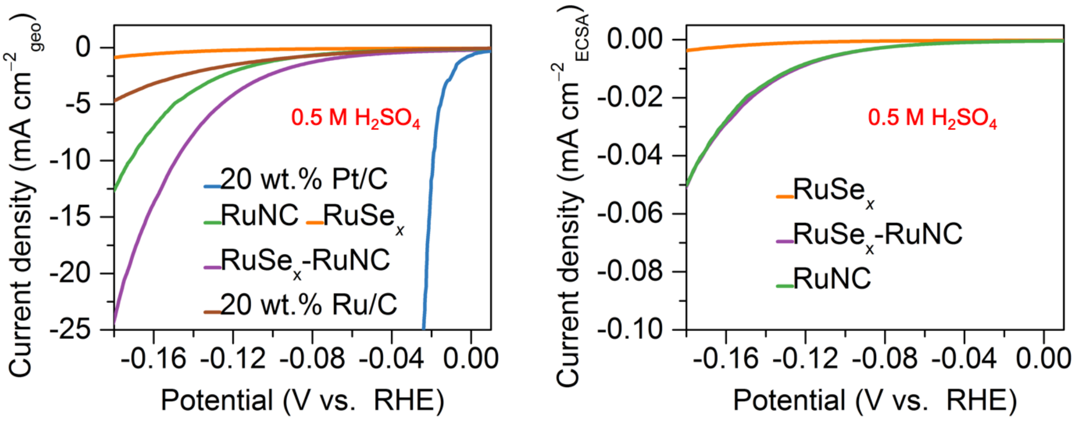

Supplementary Fig. 38 The linear sweep voltammetry curve per geometric area and the linear sweep voltammetry curve per ECSA in acidic media.

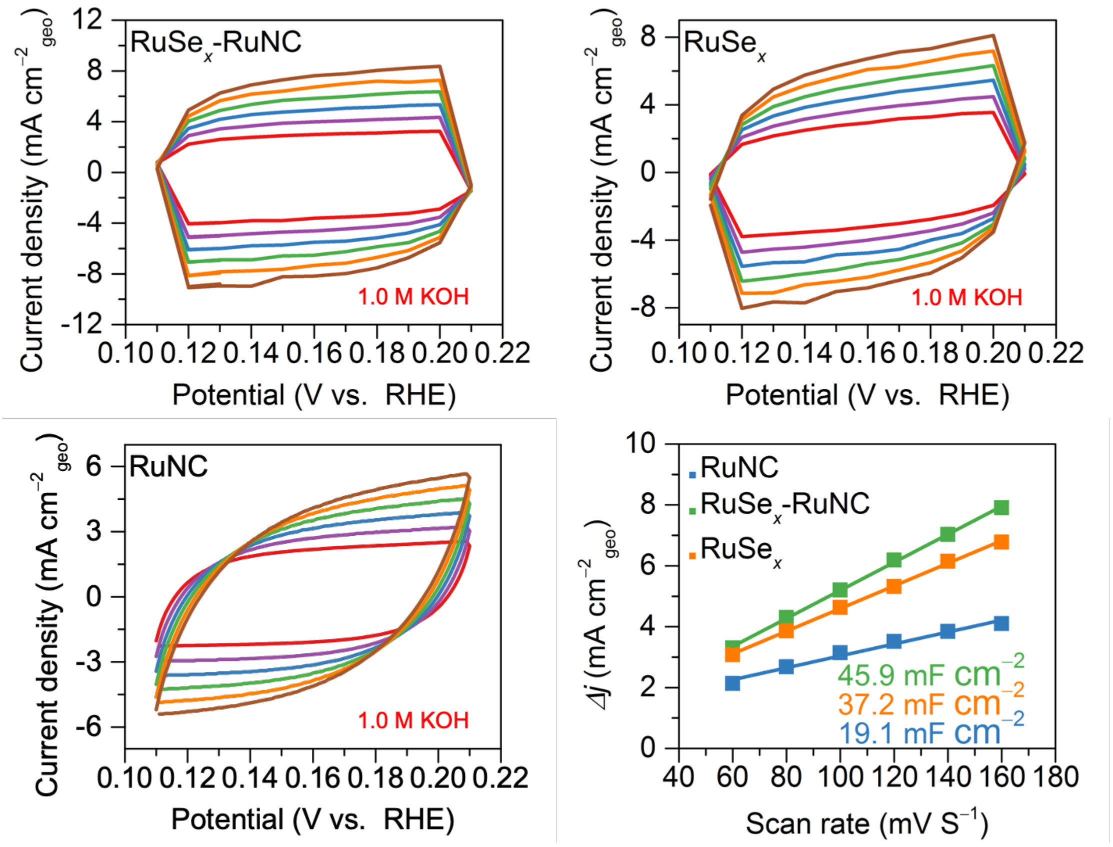

Supplementary Fig. 39 Cyclic voltammetry measured from 60 to 160 mV s^{-1} of RuSe$_x$-RuNC, RuSe$_x$, and RuNC under alkaline media and the corresponding Δj vs. scan rates plots.

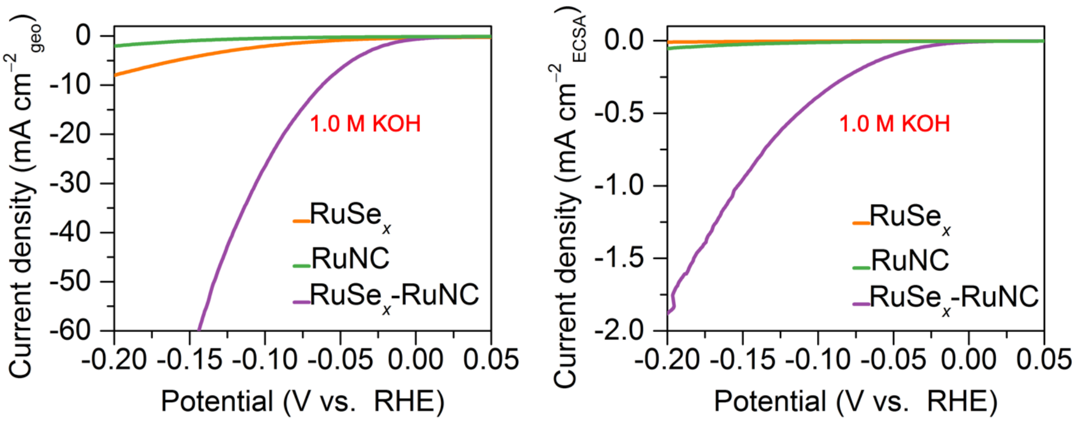

Supplementary Fig. 40 The linear sweep voltammetry curve per geometric area and the linear sweep voltammetry curve per ECSA in alkaline media.

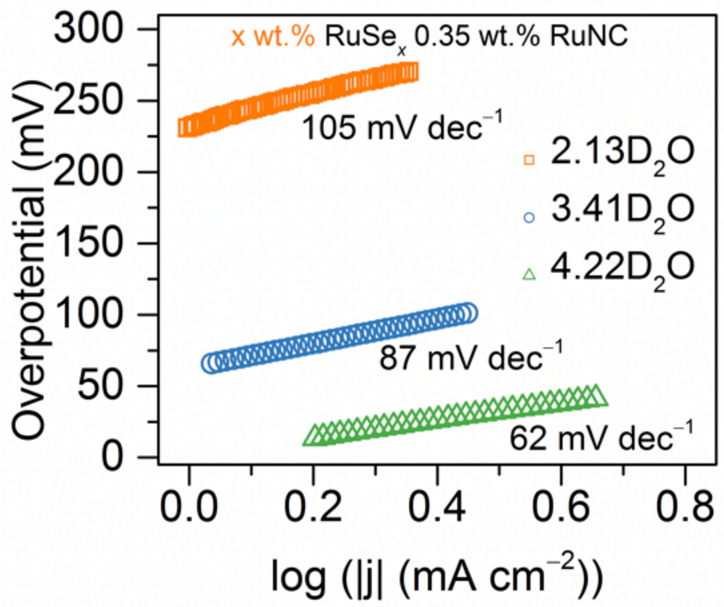

Supplementary Fig. 41 The corresponding Tafel slopes in D_2O solution.

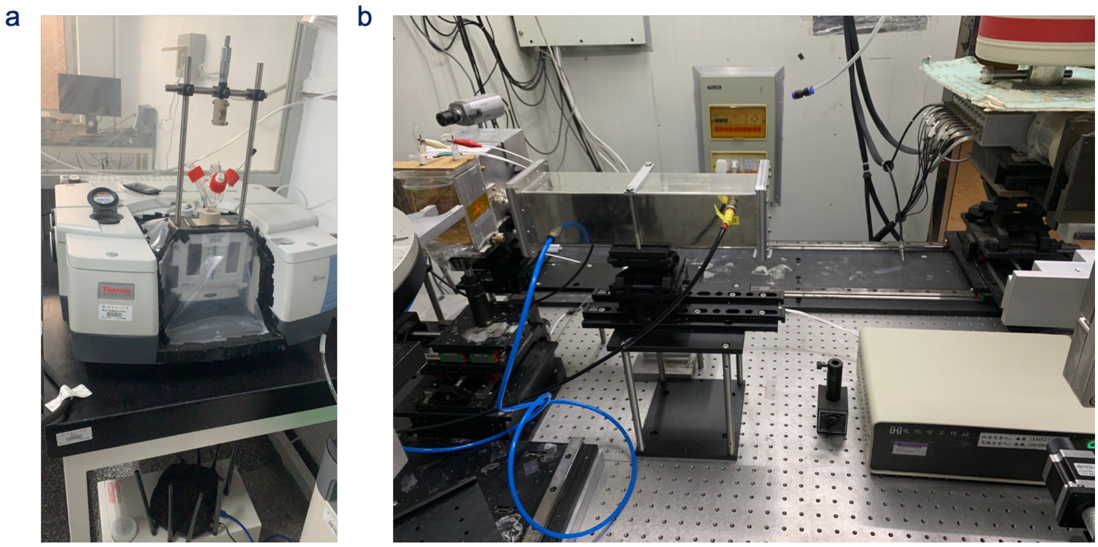

Supplementary Fig. 42. a The home-made ATR-SEIRAS test equipment. **b** The home-made XAS test equipment.

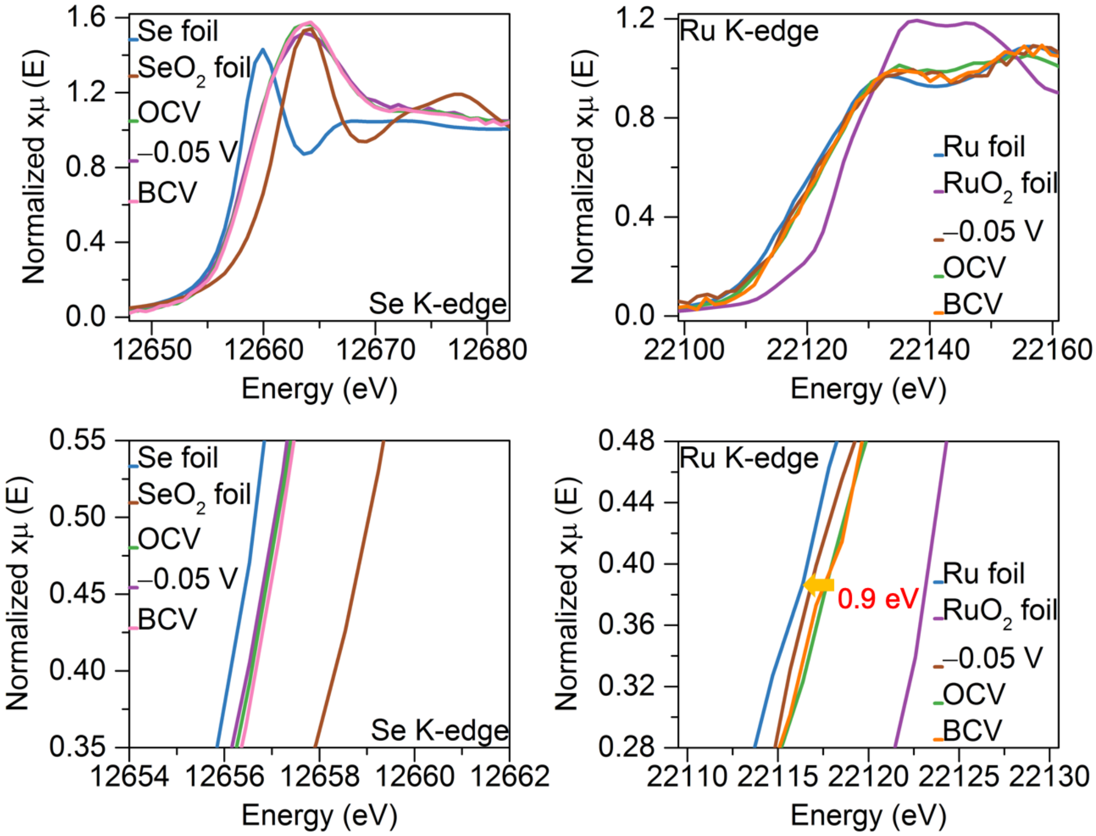

Supplementary Fig. 43 The operando Se K-edge and Ru K-edge XANES spectra.

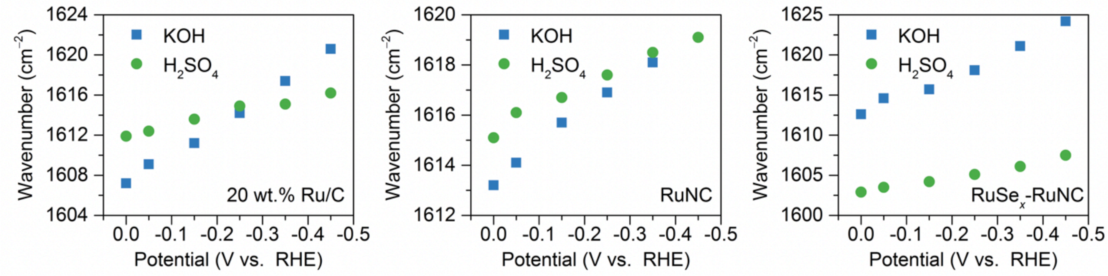

Supplementary Fig. 44 The vibrational Stark effect for the $\delta_{H\text{–}O\text{–}H}$.

2. Supplementary Tables

Supplementary Table 1 The Ru content determined by the ICP-OES analysis for the as-prepared RuNC with different $C_{15}H_{21}O_6Ru$ addition.

	0.12 wt.% RuNC	0.24 wt.% RuNC	0.35 wt.% RuNC
$C_{15}H_{21}O_6Ru$ (mg)	0.2	0.3	0.4
Ru(wt.%)	0.12	0.24	0.35

Supplementary Table 2 The Ru content determined by the ICP-OES analysis for the as-prepared $RuSe_x$ with different $C_6H_9O_6Ru$ addition.

	2.13 wt.% $RuSe_x$	3.41 wt.% $RuSe_x$	4.22 wt.% $RuSe_x$
$C_6H_9O_6Ru$ (mg)	15	20	25
Ru(wt.%)	2.13	3.41	4.22

Supplementary Table 3 Comparison of HER activity with that of previously reported electrocatalysts.

Samples	Overpotential at 10 mA•cm^{-2}	References
$RuSe_x$-RuNC	29	*This work*
MoP 700	196	*ACS Catal.* **9**, 8712–8718 (2019)
Ni0.1Co0.9P	125	*Angew. Chem. Int. Ed.* **59**, 15445–15449 (2018)
S-NiFe2O4/NF	197	*Nano Energy* **40**, 264–273 (2017)
karst NF	110	*Energy Environ. Sci.* **13**, 174–182 (2020)
CrOx/Cu–Ni	48	*Nature Energy* **4**, 107–114 (2019)
RuCo@NC	95	*Adv. Mater.* **26**, 1800047 (2018)
CoP/Co-MOF	49	*Angew. Chem. Int. Ed.* **131**, 4727–4732 (2019)
Ru@CN-0.16	100	*Energy Environ. Sci.* **11**, 800–806 (2018)
Cu-CoP NAs/CP	81	*Appl. Catal. B: Environ.* **265**, 118555 (2020)
NiCo2Px	63	*Adv. Mater.* **29**, 1605502 (2017)
RuP2@NPC	57	*Angew. Chem. Int. Ed.* **56**, 11559–11564 (2017)
Ni2P@NPCNFs	183.5	*Angew. Chem. Int. Ed.* **130**, 1981–1985 (2018)
Ni0.89Co0.11Se2 MNSN/NF	82	*Adv. Mater.* **29**, 1606521 (2017)
MoP/CNT-700	102	*Adv. Funct. Mater.* **28**, 1706523 (2018)
N-Ni	64	*J. Am. Chem. Soc.* **139**, 12283–12290 (2017)
N–Co2P/CC	42	*ACS Catal.* **9**, 3744–3752 (2019)
Co0.31Mo1.69C/MXene/NC	126	*Adv. Energy Mater.* **9**, 1901333 (2019)

Supplementary Table 4 Current, H_2 content, and Faradaic Efficiency at different potentials.

Potentials	–0.02 V	–0.04 V	–0.06 V	–0.08 V	–0.10 V
Current (mA)	0.64	1.33	2.36	3.69	5.35
H_2 (ppm)	241	504	896	1397	2019
Faradaic efficiency (%)	98.9	99.6	99.7	99.5	99.6

3. Supplementary References

1. Cherevko, S., Geiger, S., Kasian, O., Kulyk, N., Grote, J. P., Savan, A., Shrestha, B. R., Merzlikin, S., Breitbach, B., Ludwig, A., Mayrhofera, K. J. J. Oxygen and hydrogen evolution reactions on Ru, RuO_2, Ir, and IrO_2 thin film electrodes in acidic and alkaline electrolytes: A comparative study on activity and stability. *Catal. Today*. **262**, 170–180 (2016).

2. Cherevko, S. Electrochemical dissolution of noble metals native oxides. *J. Electroanal. Chem.* **787**, 11–13 (2017).
